# Identification of a novel protein complex essential for effector translocation across the parasitophorous vacuole membrane of *Toxoplasma gondii*

**DOI:** 10.1371/journal.ppat.1006828

**Published:** 2018-01-22

**Authors:** Nicole D. Marino, Michael W. Panas, Magdalena Franco, Terence C. Theisen, Adit Naor, Suchita Rastogi, Kerry R. Buchholz, Hernan A. Lorenzi, John C. Boothroyd

**Affiliations:** 1 Department of Microbiology and Immunology, Stanford University School of Medicine, Stanford, California, United States of America; 2 Department of Infectious Diseases, J. Craig Venter Institute, Rockville, Maryland, United States of America; Johns Hopkins School of Public Health, UNITED STATES

## Abstract

*Toxoplasma gondii* is an obligate intracellular parasite that can infect virtually all nucleated cells in warm-blooded animals. The ability of *Toxoplasma* tachyzoites to infect and successfully manipulate its host is dependent on its ability to transport “GRA” proteins that originate in unique secretory organelles called dense granules into the host cell in which they reside. GRAs have diverse roles in *Toxoplasma*’s intracellular lifecycle, including co-opting crucial host cell functions and proteins, such as the cell cycle, c-Myc and p38 MAP kinase. Some of these GRA proteins, such as GRA16 and GRA24, are secreted into the parasitophorous vacuole (PV) within which *Toxoplasma* replicates and are transported across the PV membrane (PVM) into the host cell, but the translocation process and its machinery are not well understood. We previously showed that TgMYR1, which is cleaved by TgASP5 into two fragments, localizes to the PVM and is essential for GRA transport into the host cell. To identify additional proteins necessary for effector transport, we screened *Toxoplasma* mutants defective in c-Myc up-regulation for their ability to export GRA16 and GRA24 to the host cell nucleus. Here we report that novel proteins MYR2 and MYR3 play a crucial role in translocation of a subset of GRAs into the host cell. MYR2 and MYR3 are secreted into the PV space and co-localize with PV membranes and MYR1. Consistent with their predicted transmembrane domains, all three proteins are membrane-associated, and MYR3, but not MYR2, stably associates with MYR1, whose N- and C-terminal fragments are disulfide-linked. We further show that fusing intrinsically disordered effectors to a structured DHFR domain blocks the transport of other effectors, consistent with a translocon-based model of effector transport. Overall, these results reveal a novel complex at the PVM that is essential for effector translocation into the host cell.

## Author summary

*Toxoplasma gondii* is an intracellular parasite capable of infecting virtually all warm-blooded animals, including humans. *Toxoplasma’s* ability to manipulate and persist in the host is due in part to its secretion of effector proteins into the host cell. Some of these effector proteins, such as GRA16 and GRA24, must cross the vacuolar membrane that the parasite replicates within during intracellular infection to reach the host cell cytosol; however, the proteins that mediate this effector transport are largely unknown. We previously identified a cleaved protein, MYR1, that is essential for effector transport. To identify other proteins that are involved in this process, we screened *Toxoplasma* mutants that are unable to induce c-Myc up-regulation in the host for their ability to transport GRA16 and GRA24 across the vacuolar membrane. Here we identify two novel proteins, MYR2 and MYR3, that are essential for effector transport. These proteins co-localize with MYR1 in the PV space and membrane and are membrane-associated. Furthermore, MYR3 stably associates with MYR1. These results indicate that MYR1 and MYR3 form a complex involved in the transport of key effectors.

## Introduction

*Toxoplasma gondii* is an obligate intracellular parasite with an extraordinary host range: it can infect virtually any nucleated cell in almost any warm-blooded animal. Upon infecting such a host, *Toxoplasma* differentiates into tachyzoites that rapidly disseminate throughout host tissues [1]. When a tachyzoite invades a host cell, it invaginates the host plasma membrane to form the parasitophorous vacuole (PV), within which it replicates during its intracellular cycle [2]. Although the vacuole protects *Toxoplasma* from clearance by the innate immune system [3, 4], it also creates a barrier that the parasite must circumvent in order to effectively hijack the host and acquire essential nutrients. *Toxoplasma*’s ability to hijack and persist in the host is due in part to its secretion of effector proteins that modulate host cell pathways, including an unknown effector responsible for up-regulation of host c-Myc [5–12]. All effector proteins so far identified within the host cell are secreted from unique organelles in the parasite, namely the rhoptries and dense granules.

Two classes of dense granule effectors have recently emerged: the first class consists of proteins, such as MAF1, GRA15, and GRA6, that localize to the nanotubular network and/or PV membrane (PVM) [5, 13, 14], while the second class consists of proteins, such as GRA16, GRA24, and TgIST, that traverse the PVM and localize to the host cell nucleus [11, 15–17]. GRA16 has been shown to affect p53 levels and PP2A-B nuclear translocation in the host. This key effector is predicted to be an intrinsically disordered protein with five nuclear localization signals and two internal repeats, one of which contains an “RRL” TEXEL (*T**oxoplasma* Export Element) motif [11]. Cleavage of the TEXEL motif by *Toxoplasma* aspartyl protease V (ASP5) is essential for GRA16 export across the PVM [18–20].

GRA24 includes an internal bipartite nuclear localization signal and two internal repeats [15]. It does not possess a TEXEL motif but is nonetheless dependent on ASP5 for its export [18–20]. GRA24 is also predicted to be an intrinsically disordered protein that interacts directly with p38α MAP kinase via a kinase-interacting motif (KIM) [15, 21]. This interaction results in the autophosphorylation of p38 MAPK and consequent activation of multiple host pathways, including upregulation of IL-12 and other pro-inflammatory cytokines.

Although it is clear that GRA16 and GRA24 must cross the PVM to ultimately reach the host nucleus, how this happens has not yet been elucidated. In *Plasmodium falciparum*, effector translocation across the PVM has been shown to depend on the PTEX complex, which consists of an HSP101 AAA+ ATPase, EXP2, PTEX150, PTEX88, and a thioredoxin domain-containing protein, TRX2 [22–24]. PTEX substrates that are fused to dihydrofolate reductase (DHFR) are still translocated into the erythrocyte cytosol; however, the addition of small molecules that stabilize the DHFR domain and prevent its unfolding effectively blocks transport, suggesting that substrates must be unfolded for translocation [25]. Consistent with this, knockdown of HSP101 AAA+ ATPase abrogates effector translocation [22, 24].

In *Toxoplasma*, the protein and molecular components required for translocation across the PVM are not well understood. GRA17 and GRA23 form pores in the PVM that allow the diffusion of small molecules [26], but these pores are not capable of translocating polypeptides and proteins. Using a mutagenesis screen, we recently identified a novel protein, MYR1, which is essential for effector translocation across the PVM and, thus, for parasite virulence in a mouse model [27]. This protein seemed unlikely to act alone, however, and so we expanded our screen to identify other proteins that are essential for effector translocation. Here we report the identification of two novel membrane proteins, MYR2 and MYR3, that are involved in effector translocation. Both proteins are found within the PV, and MYR3 stably associates with the previously identified MYR1 to form a novel complex. Furthermore, we show that the fusion of intrinsically disordered GRA16 to murine DHFR is sufficient to block its translocation, and this block affects the translocation of other effectors, which is consistent with a translocon model of effector transport.

## Methods and materials

### Parasite culture

*Toxoplasma gondii* RH*Δhpt* and RH*Δku80Δhpt* [28] strains were used for this study. *Toxoplasma* tachyzoites were maintained by serial passage in human foreskin fibroblasts (HFFs) cultured in complete Dulbecco’s Modified Eagle Medium (cDMEM) supplemented with 10% heat-inactivated fetal bovine serum (FBS), 2 mM L-glutamine, 100 U/ml penicillin and 100 μg/ml streptomycin and grown at 37 °C in 5% CO_2_. The HFFs were obtained from the neonatal clinic at Stanford University following routine circumcisions that are performed at the request of the parents for cultural, health or other personal medical reasons (i.e., not in any way related to research). These foreskins, which would otherwise be discarded, are fully de-identified and therefore do not constitute “human subjects research”. Infections included in this study were performed by scraping infected monolayers and lysing the host cells open using a 27-gauge needle. The released parasites were spun down at 1500 rpm for 5–10 min, resuspended, counted using a hemocytometer, and added to confluent HFFs at the multiplicity of infection (MOI) stated.

### Genome sequencing

For whole genome sequencing on the parental RH strain (SRR2068658), MFM1.6 (SRR2068655), MFM1.7 (SRR2068656) and MFM1.8 (SRR2068657) mutants, a single Illumina PE barcoded library was prepared from tachyzoite genomic DNA (gDNA). Libraries were then pooled into groups of nine samples and sequenced multiplexed in a single lane of an Illumina HiSeq 2000 machine to generate about 3 Gb of sequencing data per sample. As the mutants were made using the Type I RH strain as the parent, the sequencing reads were first quality trimmed with trimommatic and then mapped to the reference assembly of the Type I GT1 strain (as present in ToxoDB v13.0) with *bowtie2* [29]. After removing duplicated reads with *picard* and adjusting alignments around indels with *GATK toolkit* [30], single nucleotide variants (SNVs) were called using samtools utility *mpileup* [31] requiring a minimum base coverage of 5 reads and an alternative allele frequency of at least 80% or higher. Following this, *SnpEff* [32] together with a gff3 annotation file from the reference GT1 strain (ToxoDB v13.0) were used to classify the different types of SNVs present in each mutant. Potential change-of-function SNVs that were different between any of the three mutants and both the parental and reference strains were selected for further quality control and analysis.

### Transfections

All transfections were performed using the BTX EMC600 Electroporation System (Harvard Apparatus) or Amaxa 4D Nucleofector (Lonza) model. Tachyzoites were mechanically released in PBS, pelleted, and resuspended in solution for transfection. After transfection, parasites were allowed to infect HFFs in DMEM. Transfections with the BTX EMC600 model were performed using 5–10 x 10^6^ parasites and 15–25 μg DNA in Cytomix (10 mM KPO_4_, pH 7.6, 120 mM KCl, 5 mM MgCl_2_, 25 mM HEPES, 2 mM EDTA, 150 μM CaCl_2_). Transfections with the Amaxa 4D model were performed using 2–4 x 10^6^ parasites in 20 μl P3 solution or 5–10 x 10^6^ parasites in 100 μl P3 solution with 5–10 μg DNA. Effector translocation assays were performed by transiently transfecting pHTU-GRA24-3xMyc or pGRA1-GRA16-HA plasmid (S1 File has a list of all primers, plasmids, and strains used in this study) into tachyzoites, infecting monolayers of HFFs in DMEM, and fixing monolayers with formaldehyde at 16–24 hpi. The pGRA1-GRA16-HA plasmid, which expresses HA-tagged GRA16 off the *GRA1* promoter, was generated by amplifying *GRA16* from RH genomic DNA by PCR and cloning it into the NsiI and NcoI sites of pGRA-HPT-HA [6].

### Generation of polyclonal anti-MYR1(C) antibodies

N-terminal glutathione *S*-transferase (GST)-fused proteins were expressed using the pGEX-6P1 plasmid (Agilent Technologies). A portion of the C-terminal exon region of *MYR1* was amplified using the primers F1 and R1 (corresponding to amino acids 582–693, inclusive) and cloned into the NotI and EcoRI sites of pGEX-6P1 to generate the pGEX 6P-1 MYR1[C]-A plasmid. The resulting recombinant protein was purified from *Escherichia coli* (Rosetta strain; Novagen/EMD Millipore) as previously described [33] and injected intraperitoneally into female BALB/c (Charles River) mice with Sigma Adjuvant System (Sigma), according to the manufacturer’s instructions. Blood of naive mice was drawn prior to injection and those mice with sera that exhibited the lowest baseline reactivity against lysates and fixed monolayers of *Toxoplasma*-infected cells were selected for antibody generation. Mice were injected intraperitoneally (i.p.) with an initial dose of 100 μg protein/mouse followed by 50 μg protein/mouse boosts approximately every three weeks. Serum from these mice was isolated 14 days after each boost injection and tested on parasite lysates by Western blotting. All animal experiments were conducted with the approval and oversight of the Institutional Animal Care and Use Committee at Stanford University.

### Immunofluorescence microscopy

Infected cells grown on glass coverslips were fixed using methanol at -20°C for 20 min or 3–4% formaldehyde at room temperature (RT) for 15 min, as stated in the text. Methanol-fixed samples were washed three times for 5 min with PBS and blocked using 3% BSA in PBS for 1h at RT. Formaldehyde-fixed samples were rinsed once with PBS, permeabilized with 0.2% Triton-X 100 (TTX-100) for 20 min, and then blocked as described above. MYR1(N) protein was detected with mouse anti-MYR1 antibodies [27], while MYR1(C) protein was detected with rat anti-HA antibodies or mouse anti-MYR1 primary antibodies. GRA7 protein was detected using rabbit anti-GRA7 antibodies [34]. GRA16-HA (and other HA-tagged proteins) was detected using rat anti-HA antibodies (Roche) while GRA24-Myc was detected using rabbit anti-Myc tag antibodies (Cell Signaling Technologies). Primary antibodies were detected with goat polyclonal Alexa Fluor-conjugated secondary antibodies (Invitrogen). Vectashield with DAPI stain (Vector Laboratories) was used to mount the coverslips on slides. Fluorescence was detected using a LSM710 inverted confocal microscope (Zeiss) or epifluorescence microscope, as stated in the text. Images were analyzed using ImageJ. All images shown for any given condition/staining in any given comparison/dataset were obtained using identical parameters.

### Gene disruption

The RH*Δmyr2*, RH*Δmyr3*, and RH*Δgra16* strains were generated by disrupting the corresponding gene loci using CRISPR-Cas9 and selecting for integration of a linearized vector encoding hypoxanthine-guanine phosphoribosyltransferase (*HXGPRT)* using drug selection. Specifically, the pSAG1:U6-Cas9:sgUPRT vector [35] was modified by Q5 site-directed mutagenesis (NEB) to specify sgRNAs targeting exons in *MYR2* (F2) or *MYR3* (F3) or *GRA16* (F19). The resulting sgRNA plasmids, dubbed pSAG1:U6-Cas9:sgMYR2 and pSAG1:U6-Cas9:sgMYR3, respectively, were transfected into the RH*Δhpt* strain of *Toxoplasma* with pGRA-HPT-HA plasmid that had been linearized with NotI. The RH*Δgra16* strain was generated by co-transfecting in the pSAG1:U6-Cas9:sgGRA16 and pTKO2C mCherry vector. The parasites were allowed to infect HFFs in 24-well plates for 24 h, after which the media was changed to complete DMEM supplemented with 50 μg/ml mycophenolic acid (MPA) and 50 μg/ml xanthine (XAN) for HXGPRT selection. The parasites were passed twice into 24-well plates before being single cloned into 96-well plates by limiting dilution. Disruption of the gene coding regions was confirmed by PCR (*MYR2*: F4, R4; *MYR3*: F5, R5; *GRA16*: F20, R20) and sequencing of the locus.

### Endogenous tagging

The RH:*MYR1-3xHA* strain was generated by transfecting the pTKO_2_-MYR1-3085-3xHA vector into RH*Δku80Δhpt* and selecting for integration with MPA and Xan. The pTKO_2_-MYR1-3085-3xHA plasmid is derived from the pTKO_2_-HPT-3xHA vector, which was generated by cloning 3xHA tag amplified from synthesized DNA (IDT) into the EcoRV and EcoRI sites of the previously described pTKO2C mCherry vector [36]. Approximately 3,000 bp of the *MYR1* 3’ coding sequence and HA tag were amplified from RH genomic DNA using the F8 and R8 primers, and the resulting insert was cloned into the NotI and EcoRV sites of the pTKO_2_-HPT-3xHA vector. The plasmid was linearized at the SmaI site in the insert, and 25 μg of the linearized plasmid was transfected by electroporation. Parasites were allowed to infect HFFs in T25 flasks for 24 h, after which the medium was changed to complete DMEM supplemented with 50 μg/ml MPA and 50 μg/ml XAN for HXGPRT selection. Parasites were passaged twice before being singly cloned into 96-well plates by limiting dilution. Screening for correct integration into the endogenous locus was performed by PCR using multiple primers, including F9 and R9.

The RH:*MYR2-3xHA* strain was generated by transfecting the pTKO2-MYR2-1151-3xHA vector into RH*Δku80Δhpt* and selecting for integration with MPA/XAN. The pTKO2-MYR2-1151-3xHA vector was constructed by amplifying approximately 1500 bp of the *MYR2* 3’ coding sequence with F10 and R10 primers and cloning it into the NotI and EcoRV sites of the pTKO2-HPT-3xHA vector. The construct was then linearized with Bsu361, and the linearized plasmid was transfected as described above. The RH:*MYR2-3xHA*_*62*_ strain was generated by amplifying (F11, R11) a 740 bp gBlock (IDT) in which *MYR2* encodes a 3xHA tag starting at amino acid 62 and co-transfecting the purified product with pSAG1:U6-Cas9:sgMYR2-178 vector (F13) into RH*Δku80Δhpt* to create a double stranded break. The tag in the gene block is positioned to disrupt the PAM motif of the sgRNA target, which prevents targeting of the gBlock or integrated sequence.

The RH:*MYR3-3xHA* strain was generated by amplifying approximately 700 bp of *MYR3* 3’ coding and UTR sequence (F12, R12) synthesized with a C-terminal 3xHA tag (IDT) and co-transfecting it into RH*Δku80Δhpt* with pSAG1:U6-Cas9:sgMYR3-C2 plasmid (F14). Parasites transiently expressing Cas9-GFP were enriched by FACS sorting at 24 hours post-infection, and the resulting population was allowed to infect HFFs in DMEM. The parasites were cloned by limiting dilution and screened for 3xHA integration at the *MYR3* locus by PCR (F15 and R15) and sequencing.

### Endogenous mutation

The RH:*MYR1 R577A* strain was generated by amplifying (F16, R16) a gBlock (IDT) in which the codon for arginine at residue 577 of MYR1 is mutated to one for alanine. The resulting product was purified and co-transfected into RH:*MYR1-HA* [27] with pSAG1:U6-Cas9:sgMYR1 2081. Transfected parasites were enriched by FACS sorting and cloned by limiting dilution, as described above.

### Ectopic gene integration

The RH*Δmyr3*::*MYR3* strain was complemented ectopically with the pGRA-MYR3-3xHA plasmid, which expresses *MYR3* off its natural promoter. To construct the pGRA-MYR3-3xHA plasmid, the *MYR3* promoter and open reading frame were amplified from RH*Δhpt* genomic DNA using F6 and R6 primers. The resulting ~2 kb fragment was digested with HindIII and NcoI restriction enzymes and cloned into the corresponding sites of the pGRA1-HPT-3xHA backbone. The resulting vector, pGRA-MYR3-3xHA, was linearized with NotI, and 5 μg of the linearized plasmid was co-transfected with 5 μg pSAG1:U6-Cas9:sgUPRT [35] into RH*Δmyr3* tachyzoites. Integration of the vector at the *UPRT* locus was enriched by selecting for resistance to 5 μM FUDR in DMEM after one lytic cycle. The resulting population was then cloned by limiting dilution and tested for MYR3-3xHA expression by Western blot and IFA.

To construct the pGRA1-HPT-3xHA vector, a 296 bp double stranded DNA gene block (IDT) was designed to contain the sequence of the original pGRA1-HPT-HA plasmid from the NsiI site to the NcoI site, a triple HA tag and stop codon after the NcoI site, and a PacI site after the stop codon. The gene block was amplified, digested with NsiI and PacI restriction enzymes, and cloned into the corresponding sites of the pGRA1-HPT-HA plasmid.

The RH*Δmyr2* strain was complemented ectopically by transient transfection with the pGRA1-MYR2-3xHA plasmid, which expresses C-terminally tagged MYR2 off the *GRA1* promoter. The pGRA1-MYR2-3xHA plasmid is derived from the pGRA1_plus_-HPT-3xHA plasmid, which is derived from pGRA1-HPT-3xHA plasmid and includes two additional restriction sites and an extended *GRA1* promoter. Approximately 2 kb of the *MYR2* open reading frame (ORF) was amplified from RH genomic DNA using F7 and R7 primers and cloned into the pGRA1 NsiI and NcoI restriction sites of pGRA1 _plus_-HPT-3xHA by Gibson Assembly (NEB). To assess complementation, 5 μg pGRA1-MYR2-3xHA plasmid was co-transfected with 5 μg GRA24-3xMyc plasmid, and effector translocation was assessed by IFA.

The RH:*MYR1-3xHA*::*GRA16-DHFR* strain was generated by co-transfecting linearized pGRA1:GRA16-DHFR plasmid and pSAG1:U6-Cas9:sgUPRT plasmid into the RH:*MYR1-3xHA* strain and enriching for integration at the *UPRT* locus with FUDR, as described above. The pGRA1:GRA16-DHFR plasmid was generated by amplifying murine DHFR coding sequence from a gBlock (IDT) and cloning it into the NcoI and PacI sites of pGRA1:GRA16-HA, effectively replacing the HA tag with mDHFR. The pGRA1-GRA16-DHFR plasmid was linearized with NotI for transfection. The RH::*MYR3-3xHA* strain was made by transfecting RH*Δhpt* with pGRA-MYR3-3xHA and selecting for integration with MPA/XAN, as described above. The RH*Δmyr2*::*MYR3-3xHA* strain was made by co-transfecting RH*Δmyr2* with pGRA-MYR3-3xHA and pSAG1:U6-Cas9:sgUPRT and selecting for UPRT disruption with FUDR, as described above.

### Fractionation

Tachyzoites were allowed to infect HFF monolayers for 20–24 h in DMEM. Infected monolayers were washed, mechanically disrupted in PBS with HALT protease and phosphatase inhibitors (Thermo Fisher) using a 27-gauge syringe, and spun down at 2500 x g to yield the low speed supernatant (LSS) and low-speed pellet (LSP), the latter of which consists of parasites and large host cell debris. The LSP was resuspended in cell extraction buffer (Invitrogen) supplemented with HALT inhibitors, incubated on ice, spun at 20,000 x g to pellet nuclear debris, and the resulting supernatant was then saved at -20°C for analysis. The LSS fraction containing PV membranes and soluble material was further fractionated at 100,000 x g at 4°C for 1 h to yield the high-speed pellet (HSP) and high-speed supernatant (HSS). The HSP was treated with PBS or 1% TTX-100 plus 0.1% sodium dodecyl sulfate (SDS) for 30–45 min on ice, and fractionated again at 100,000 x g at 4°C for 1 h. After the final centrifugation, HSP fractions were resuspended in cell extraction buffer (Invitrogen), boiled in SDS sample buffer, and disrupted with a syringe for SDS-PAGE. HSS fractions were concentrated using TCA precipitation or centrifugal filters (Cat#: XUFC201024, EMD Millipore) and boiled in sample buffer. To assess MYR3 phosphorylation, monolayers infected with RH:*MYR3-3xHA* tachyzoites were washed, mechanically disrupted, and fractionated as described above in PBS containing an EDTA-free protease inhibitor cocktail (Roche) plus or minus PhosStop phosphatase inhibitors (Roche).

### Western blotting

Cell lysates were prepared at 20–24 hours post-infection (hpi) in cell extraction buffer (Invitrogen) plus protease and phosphatase inhibitors (Roche, Thermo Fisher). Samples containing 10–20 μg protein were boiled for 10 min in sample buffer, separated by SDS-PAGE, and transferred to polyvinylidene difluoride (PVDF) membranes. Membranes were blocked in 5% milk in TBS supplemented with 2.5% Tween-20 for 1 h at RT and then incubated overnight at 4°C with primary antibody in blocking buffer. HA was detected using horseradish peroxidase (HRP)-conjugated HA antibody (Roche). MAF1 was detected using rabbit anti-MAF1 antibodies [13] and GRA7 protein was detected using rabbit anti-GRA7 antibodies [34]; both were detected using goat anti-rabbit IgG (H+L)-HRP conjugate. MYR1(C) and (N) terminal fragments were detected using mouse anti-MYR1 antibodies, while GRA1 was detected using mouse anti-GRA1 antibodies (Biotem); these antibodies were detected with goat anti-mouse IgG (H+L)-HRP conjugate. Horseradish peroxidase (HRP) was detected using enhanced chemiluminescence (ECL) kit (Pierce). Membranes were stripped between blots by incubation in stripping buffer (Thermo Fisher) for 10–15 min then washed 2 x 5 min with PBS or TBS-T.

### Immunoprecipitations

Confluent monolayers of HFFs in 15-cm dishes were infected at MOI 1–2 with 3xHA-tagged parasites or untagged parental controls for 20–24 h. Infected monolayers were washed 3 times with ice-cold PBS, then scraped and mechanically disrupted with a 27-gauge syringe in lysis buffer (50 mM Tris, pH 7.6, 200 mM NaCl, 1% TTX-100, 0.5% CHAPS) supplemented with protease and phosphatase inhibitors (Roche, Thermo Fisher). The lysate was sonicated 3 x 10 sec at 50% duty cycle, output control 2 with the Branson Sonifier 250 and left on ice for 30–45 min. The samples were spun at 1200 x g to pellet out any nuclei and remaining debris, and input was collected from the resulting supernatant. The remaining supernatant was incubated with magnetic beads conjugated to anti-HA antibodies (Thermo Fisher) overnight at 4°C with gentle rotation. The beads were washed twice with lysis buffer and four times with wash buffer (50 mM Tris, pH 7.6, 300 mM NaCl, 0.1% TTX-100) using a magnetic rack. Bound peptides were eluted by incubating the beads with 2 mg/ml anti-HA peptide (Thermo Fisher) at 37°C for 15 min with gentle rotation.

### Ethics statement

This study was carried out in strict accordance with the Public Health Service Policy on Humane Care and Use of Laboratory Animals and AAALAC accreditation guidelines. The protocol was approved by Stanford University’s Administrative Panel on Laboratory Animal Care (Animal Welfare Assurance # A3213-01, protocol # 9478). All efforts were made to minimize suffering.

## Results

### Identification of genes essential for effector translocation

*Toxoplasma* mutants unable to upregulate host c-Myc were isolated using a forward genetic screen, as previously described [27]. Briefly, bone marrow macrophages (BMMs) engineered to express GFP fused to c-Myc were infected with mCherry-expressing *Toxoplasma* tachyzoites that had been chemically mutagenized with ethylnitrosourea (ENU). Successive FACS sorts were performed to enrich for BMMs that were infected with tachyzoites incapable of modulating host c-Myc regulation (i.e., had a Myr^-^ phenotype). Initial screening of these mutant populations yielded 3 independent mutants with a defect in GRA16 and GRA24 translocation across the PVM; whole genome sequencing revealed each of the 3 mutants had a distinct nonsense mutation in a gene dubbed *MYR1* (*TGGT1_254470*) [27].

**Fig 1:**
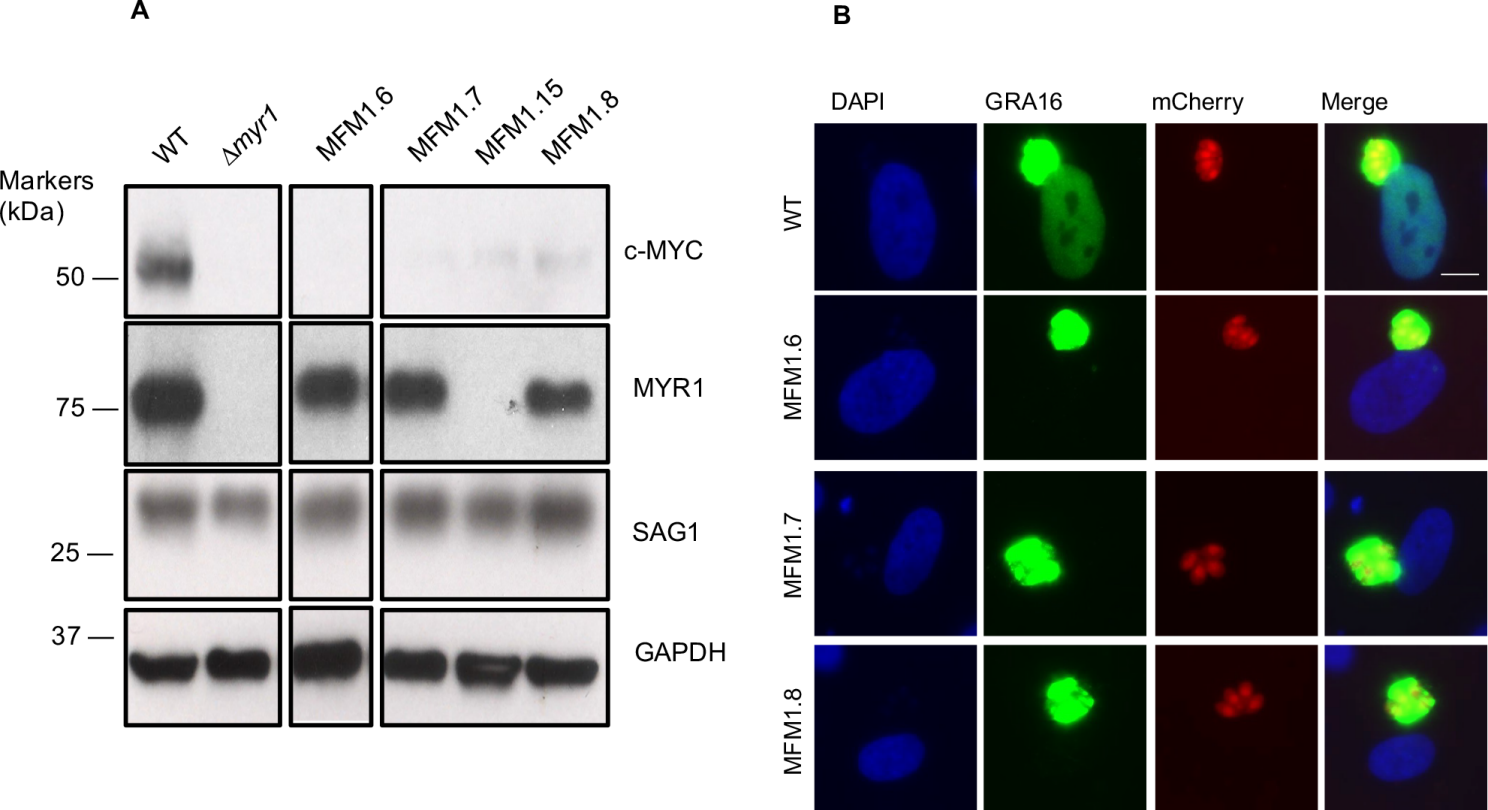
Identification of mutants defective in effector translocation across PVM. **(A)** Identification of mutants deficient in c-Myc upregulation that express full-length MYR1 protein. Lysates were generated from human foreskin fibroblasts (HFFs) infected with *Toxoplasma* mutants unable to upregulate host c-Myc. The lysates were resolved by SDS-PAGE, blotted, and probed with antibodies against MYR1(N), c-Myc, GAPDH, and SAG1. **(B)** Mutants are defective in effector translocation. Three *Toxoplasma* mutants (MFM1.6, MFM1.7, and MFM1.8) that are wild-type with respect to *MYR1* yet deficient in upregulation of host c-Myc were tested for GRA16 localization relative to wild-type control. The mCherry-expressing mutants were transiently transfected with a plasmid expressing GRA16-HA and allowed to infect HFFs. At 16 hours post-infection (hpi), infected coverslips were fixed and GRA16-HA was localized with anti-HA antibodies. DAPI was used to identify host nuclei.

The previously published work showed that MYR1 localizes to the PVM and is necessary for GRA16 and GRA24 effector translocation. Based on precedent in other protein translocation systems, however, we hypothesized that multiple proteins are involved and set out to identify these other proteins. To do this, we performed Western blots on 13 additional mutants isolated from the original Myr^-^ population to test for the expression of full-length MYR1 protein, which indicates that they may be mutated in other crucial genes. Of these 13 clones, 8 appeared wild-type for *MYR1*, indicating they likely harbored a mutation in a different gene involved in the Myr^-^ phenotype, and the presence of a wild-type *MYR1* locus was confirmed by PCR amplification and sequencing of the *MYR1* locus (Fig 1A). Three of these mutants (MFM1.6, MFM1.7, and MFM1.8) were chosen at random for whole-genome sequencing to identify the mutated genes that might underlie their Myr^-^ phenotype.

Genomic sequencing revealed the expected range of 7 to 18 coding mutations in the three mutants (Table 1). Mutant MFM1.8 had no mutation in any gene that was also mutated in MFM1.7 and MFM1.6, but it did have a nonsense mutation in gene *TGGT1_270700* (Q174*). This appeared the most likely candidate for the phenotype-causing mutation in the MFM1.8 mutant given that it was the only stop codon acquired and *TGGT1_270700* is predicted to encode a novel, secreted protein. To test this hypothesis, we amplified the *TGGT1_270700* locus in the other 5 MYR1-wild-type Myr^-^ mutants to see if any were independently mutated in this gene. We found that one such mutant, MFM1.13, had an independent nonsense mutation (Y274*) in *TGGT1_270700*. This strongly argued for *TGGT1_270700* being key to the c-Myc up-regulation phenotype. The MFM1.6 mutant also had a single nonsense mutation and this was in a gene, *TGGT_237230*, which initially appeared to be unaltered, in terms of coding mutations, in the other two sequenced mutants. On further inspection, however, it was apparent that *TGGT1_237230* was misannotated in ToxoDB and that a region originally called as an intron is in fact part of the coding region in the vast majority of transcripts. Using this corrected coding region and reanalyzing the MFM1.7 sequencing data revealed that MFM1.7 had a missense mutation in *TGGT1_237230* (1), strongly suggesting mutations in this gene are responsible for the Myr^-^ phenotype in the MFM1.6 and MFM1.7 mutants. PCR sequencing of the loci described here in the remaining 4 *MYR1* wild-type mutants indicated all were clones of MFM1.7 and so these were not further pursued.

**Table 1:** Coding mutations in mutant strains as detected by sequencing.

Chromosome	SNV position	% Reads mutant allele	Mutation (WT/mut)^a^	Amino acid change^b^	Gene	Predicted function
**MFM1.8**						
Ib	1182115	100	C/T	Glu732Lys	209270	hypothetical protein
X	6553250	100	C/T	Gly2730Asp	214990	hypothetical protein
XII	1175195	97	A/G	Thr582Ala	218890	hypothetical protein
V	111982	100	A/G	Ser993Pro	220208	hypothetical protein
VIII	5065649	97	G/A	Gln174*	270700	hypothetical protein
VIII	3385162	97	A/G	Val1202Ala	273560	kinesin heavy chain, putative
IV	912298	100	G/T	Gln869Lys	319390	hypothetical protein
**MFM1.6**						
XI	6048743	100	A/G	Ile10Thr	216470	hypothetical protein
II	6543	100	A/G	Ser10Pro	220840	threonine synthase
X	1397438	100	C/G	Thr2448Ser	226620	hypothetical protein
X	456235	100	A/T	Asn1812Ile	228150	hypothetical protein
VIII	655253	100	A/C	Ser751Arg	230170	hypothetical protein
X	5689666	100	A/C	Tyr100*	237230	hypothetical protein
VI	1867636	100	A/G	Val646Ala	242320	B-box zinc finger protein
VIIb	3737373	100	A/G	Ser833Pro	257800	adenylyltransferase
VIIb	3419925	100	G/C	Pro435Ala	258360	hypothetical protein
VIIb	3107324	100	C/G	Thr581Arg	258840	hypothetical protein
VIIb	1993725	100	G/C	Gln999His	260830	hypothetical protein
IX	1035797	100	G/T	Val216Phe	266360	hypothetical protein
VIII	3775425	100	A/G	Ser2769Pro	272710	transcription factor AP2VIII-4
XII	6715349	100	G/C	Ala53Pro	276990	cytochrome b5 family protein
IX	3005919	100	T/C	Val486Ala	289330	ubiquitin hydrolase family 2
VIIa	499697	98	T/C	Ile74Val	304490	hypothetical protein
IX	5499899	100	C/G	Leu1510Val	306000	transcription factor AP2IX-8
IV	321900	100	G/C	Gly45Arg	320410	hypothetical protein
**MFM1.7**						
X	6416305	100	C/G	Cys1296Trp	214830	hypothetical protein
VI	1806568	100	C/G	Gln163Glu	242070	kinase regulatory subunit
XII	3000477	100	T/C	Thr966Ala	246982	hypothetical protein
XII	3503224	100	G/C	Thr282Arg	247760	AMP-binding protein
IX	1938795	95	T/G	Val701Gly	264670	DNA polymerase family B protein
VIII	5386411	100	T/C	Thr63Ala	270140	splicing factor DIM1, putative
X	5689475	100	G/A	Thr164Ile^c^	237230	hypothetical protein

The nature of all coding SNVs in chromosomal genes for mutants is listed.

^a^ WT, wild-type; mut, mutant.

^b^ *, stop codon

^c^ Mutation is in miscalled intron (ToxoDB (v13.0)) and therefore not present in the original list of coding mutations. Mutation was identified by searching for mutations listed in introns in this gene and confirmed by PCR and sequencing of the locus.

Having identified novel Myr^-^ mutants, we next asked whether they are defective in effector translocation or in c-Myc up-regulation only. To test this, we independently transfected the MFM1.6, MFM1.7, and MFM1.8 mutants with a GRA16-HA plasmid and assessed effector protein localization by immunofluorescence assay (IFA). The results showed that all three mutants have a defect in the translocation of GRA16 across the PVM to the host nucleus, indicating that these mutants are indeed defective in the machinery responsible for the translocation of effectors rather than in an effector that mediates host c-Myc upregulation (Fig 1B).

**Fig 2:**
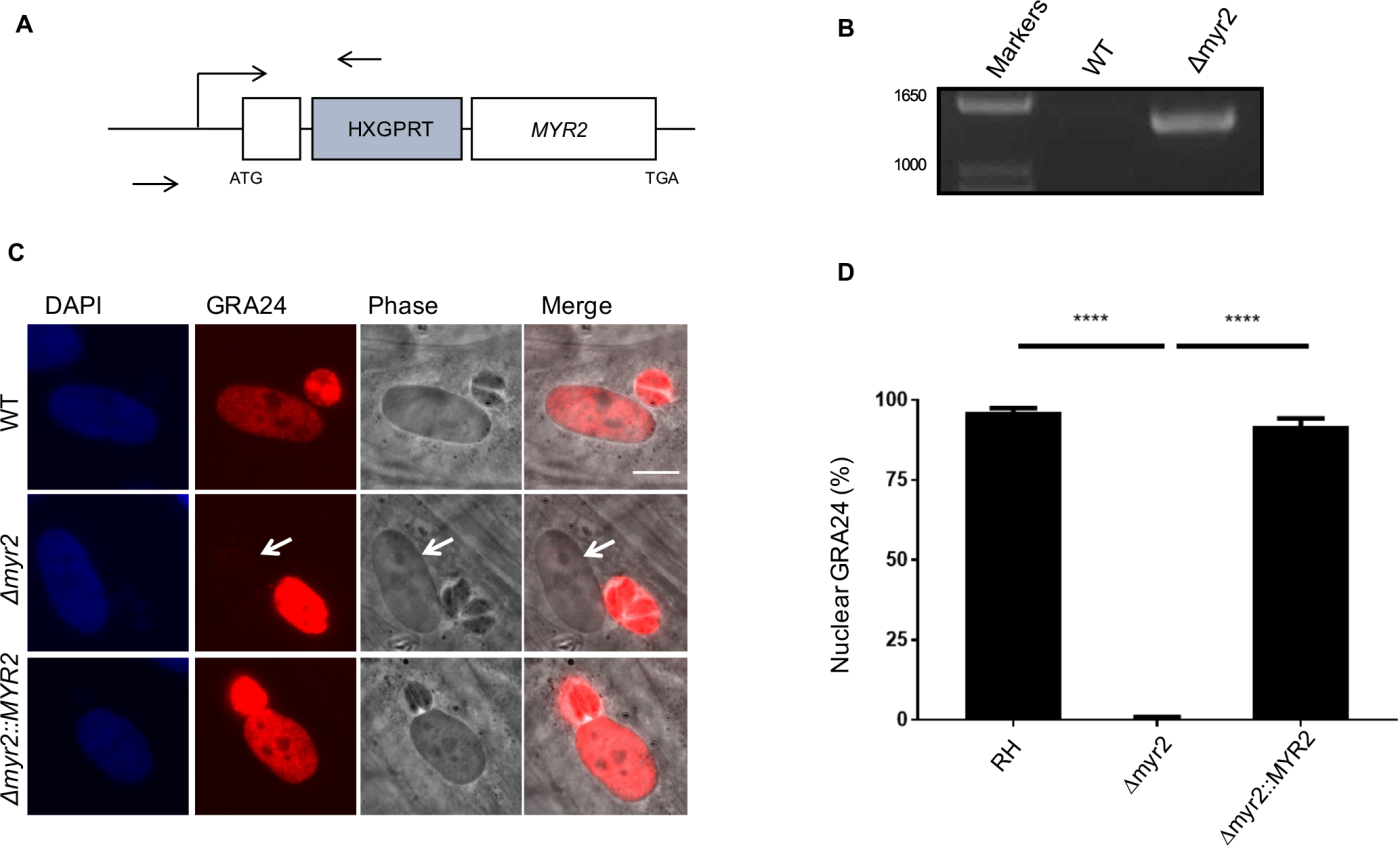
MYR2 (*TGGT1_270700*) is essential for effector translocation. **(A)** Schematic of *TGGT1_270700* (*MYR2*) disruption. To disrupt the *MYR2* locus, a CRISPR-Cas9 plasmid encoding a sgRNA against *TGGT1_270700* was co-transfected with linearized pGRA1-HA-HPT plasmid into RH*Δhpt* parasites. Arrows indicate location of primers used to confirm integration of vector in the gene locus (and consequent disruption of the gene ORF) by PCR. (**B)** Disruptive integration of the HXGPRT vector in an exon of *MYR2* was confirmed by PCR and sequencing. (**C)** Effect of *MYR2* disruption and complementation on effector translocation. Wild-type (WT) and RH*Δmyr2* tachyzoites were transiently transfected with a plasmid expressing Myc-tagged GRA24. For complementation, the *Δmyr2* strain was co-transfected with GRA24-Myc and pGRA1:MYR2-3xHA plasmids. Transfected tachyzoites were allowed to infect HFFs for 16–24 hours. The infected monolayers were then fixed and GRA24 and MYR2 localization was assessed with anti-myc tag and anti-HA antibodies, respectively. Scale bar indicates 5 μm. White arrow indicates host nucleus lacking detectable GRA24 in the cell infected with RH*Δmyr2* tachyzoites. **(D)** Quantitation of percentage of infected cells showing GRA24 localization in the host nucleus based on three independent experiments, each with analysis of 10 fields on at least three coverslips. Statistics were performed with one-way ANOVA and Tukey’s multiple comparison’s test. **** indicates P < 0.0001.

**Fig 3:**
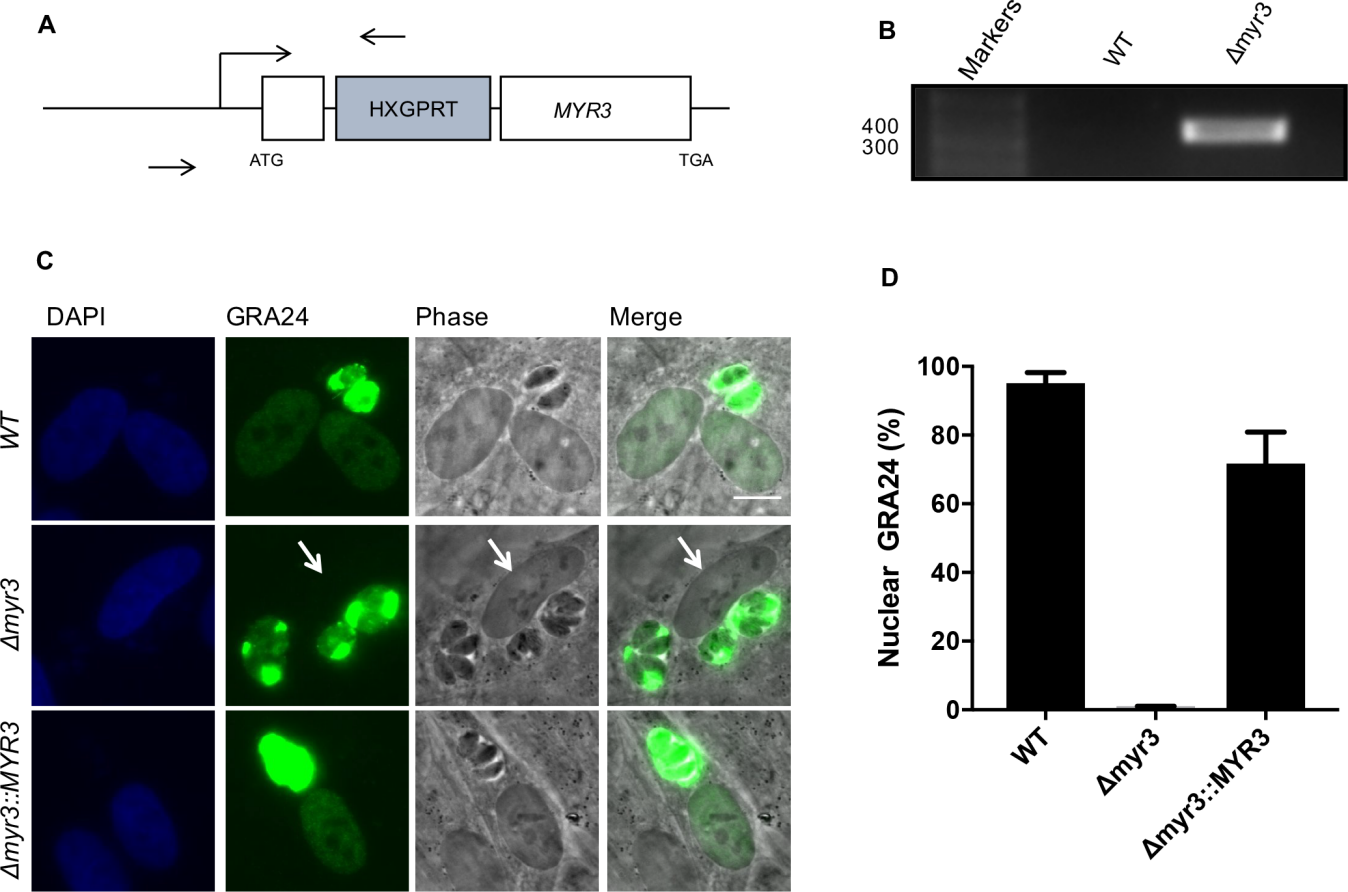
MYR3 (*TGGT1_237230*) is essential for effector translocation. **(A)** Schematic of *TGGT1_237230* (*MYR3*) disruption. MYR3 gene disruption was performed as described in (2A), but using a sgRNA and primers specific to *MYR3*. (**B)** Disruptive integration of the HXGPRT vector in an exon of *MYR3* was confirmed by PCR and sequencing. (**C)** Effect of *MYR3* disruption and complementation on effector translocation. GRA24-Myc was transfected into WT, *Δmyr3*, and *Δmyr3*::*MYR3* tachyzoites and its localization was assessed by IFA. Scale bar indicates 5 μm. White arrow indicates host nucleus lacking detectable GRA24 in the cell infected with RH*Δmyr3* tachyzoites. (**D)** Quantitation of percentage of infected cells showing GRA24 localization in the host nucleus based on examination of at least 10 fields per each of three coverslips. Error bars reflect standard deviation for three to four replicates. Results are representative of 5 experiments.

To verify that *TGGT1_270700* and *TGGT1_237230* are responsible for the Myr^-^ phenotype, we generated strains disrupted in the first exon of each gene; specifically, we co-transfected CRISPR-Cas9 sgRNA plasmid modified to target the first exon of the relevant gene along with linearized pGRA1-HA-HPT plasmid, which contains the *HXGPRT* selectable marker, into RH*Δhpt* parasites (Figs 2A and 3A). Following selection with mycophenolic acid and xanthine, we cloned the populations by limiting dilution and confirmed disruptive integration of the vector in the first exon of each gene by performing PCR (Figs 2B and 3B) and submitting the product for sequencing. To assess the role of these genes in effector translocation, we transfected the parental strain and RH*Δ270700* and RH*Δ237230* strains with GRA24-myc plasmid and assessed GRA24 localization by IFA. The results show that *TGGT1_270700* and *TGGT1_237230* are indeed essential for effector translocation, and we therefore dubbed them *MYR2* and *MYR3*, respectively (Figs 2C, 2D, 3C and 3D).

The *MYR2*-disrupted line was complemented ectopically by transient expression of MYR2-3xHA off the *GRA1* promoter along with pHTU-GRA24-Myc. We first attempted to express MYR2-3xHA off the *MYR2* promoter, but we were unable to visualize the HA-tagged protein by this method, likely due to its low protein expression, as discussed below. GRA24 localization was then assessed by IFA and compared to wild-type and RH*Δmyr2* strains transfected with the pHTU-GRA24-Myc plasmid alone. The results showed that the parental and complemented parasites had robust GRA24 localization in the vacuole and infected host cell nucleus while RH*Δmyr2* had robust signal in the vacuole but not in the host nucleus (Fig 2C and 2D). This confirmed *MYR2* as a gene necessary for protein translocation of effectors across the PVM.

RH*Δmyr3* parasites were ectopically complemented by co-transfecting the CRISPR-Cas9 construct targeting *UPRT* and a construct expressing MYR3-3xHA off its native promoter. Complemented lines harboring a disruption of *UPRT* and integration of *MYR3-3xHA* at the *UPRT* locus were enriched with FUDR selection. To confirm the importance of *MYR3* in effector translocation, we transfected the WT, RH*Δmyr3*, and RH*Δmyr3*::*MYR3* strains with a plasmid expressing GRA24-Myc and assessed GRA24 localization by IFA. The results showed a substantial defect in GRA24 nuclear localization in cells infected with RH*Δmyr3* parasites, but not parental or complemented parasites, confirming MYR3 is an essential component for effector translocation (Fig 3C and 3D).

### MYR1, MYR2, and MYR3 affect growth *in vitro*

**Fig 4:**
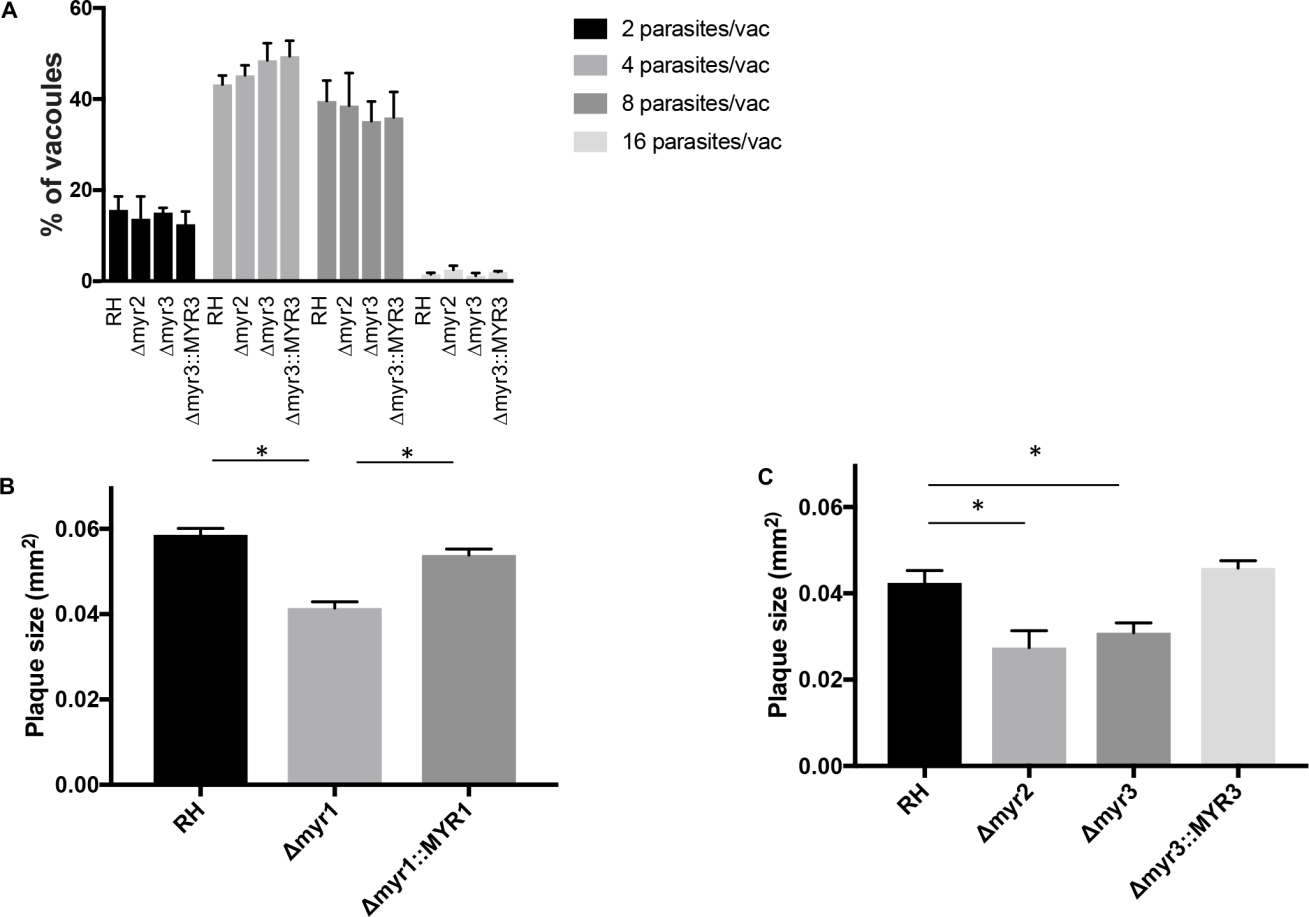
MYR1, MYR2, and MYR3 are necessary for fully efficient growth *in vitro*. (A) HFFs were infected with tachyzoites of the indicated strains for 20 hours, fixed, and then stained with antibody to SAG1 to assess the number of parasites per vacuole. The results indicate the percentage of vacuoles containing 2, 4, 8, or 16 parasites. The averages are based on 3 independent replicates, and error bars reflect SEM. (B, C) HFFs were infected with tachyzoites of the indicated strains for 6 days, fixed with methanol, and then stained with crystal violet. Plaque size was measured using ImageJ. The averages are based on results from at least 3 independent biological replicates, each with 2–3 technical replicates, and error bars indicate SEM. *p < 0.005 using one-way ANOVA and Tukey’s multiple comparison’s test.

GRA16, GRA24, and IST have not been shown to affect growth *in vitro*, and our previous work has shown that deletion of *MYR1* does not affect the number of parasites per vacuole *in vitro* 22 hours post infection (hpi). To determine if loss of *MYR2* or *MYR3* affects growth at a similarly early time point, we assessed the number of parasites per vacuole in HFFs infected with RH wild type, RH*Δmyr2*, RH*Δmyr3*, or RH*Δmyr3*::*MYR3-3xHA* tachyzoites 20 hpi. Consistent with our previous results for MYR1, we found no apparent defect in growth for RH*Δmyr2* and RH*Δmyr3* tachyzoites (Fig 4A) at 20 hpi. This is a relatively early time point, however, and so to determine if loss of *MYR1*, *MYR2*, or *MYR3* have even a subtle effect on growth, we infected HFFs with tachyzoites from these same strains, fixed the monolayers at 6 days post infection, and measured plaque size. The results showed a small but significant defect in plaque size for *Δmyr1*, *Δmyr2*, *and Δmyr3* strains, that at least in the case of *Δmyr1* and *Δmyr3* was rescued by the relevant complementation (Fig 4B and 4C; we were unable to obtain a stable complement of the *Δmyr2* mutant). This indicates that MYR1, MYR2 and MYR3 are all necessary for fully wild type growth, even *in vitro*, and suggests that one or more effectors translocated by the MYR1/2/3-dependent machinery has a role in growth in HFFs.

**Fig 5:**
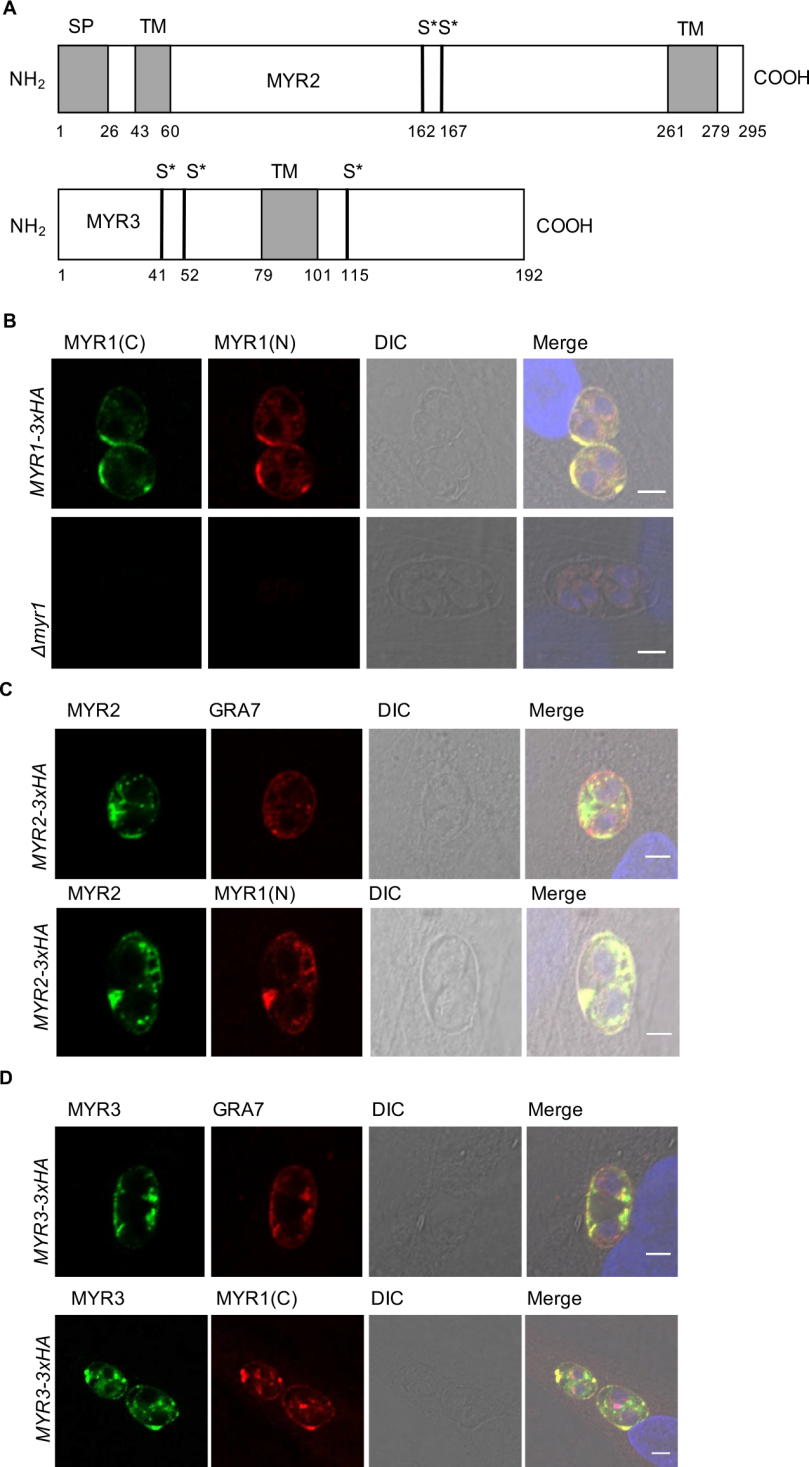
MYR1, MYR2, and MYR3 co-localize at the PV membrane and space. **(A)** MYR2 and MYR3 structure. Top: Schematic of MYR2 protein sequence with predicted signal peptide (SP), transmembrane domains (TM), and previously determined phosphorylated serine residues (S*) indicated [37]. Bottom: Similarly annotated schematic of MYR3. **(B)** Localization of MYR1(C)- and (N)-terminal fragments. RH tachyzoites stably expressing an endogenously 3xHA-tagged MYR1(C)-terminus or deleted for the *MYR1* gene (*Δmyr1*) were allowed to infect HFFs. At 12 hpi, infected monolayers were fixed with methanol. The MYR1(C)-terminus was stained with anti-HA antibodies, the MYR1 (N)-terminus was stained with antibodies to a recombinant, N-terminal fragment of MYR1, and their localization was assessed using confocal microscopy. Host nuclei were stained with DAPI and differential interference contrast (DIC) microscopy was used to visualize the monolayer. Scale bar is 5 μm. (**C)** MYR2 localization. RH*Δmyr2* tachyzoites were transiently transfected with a construct that expresses 3xHA-tagged MYR2 off the GRA1 promoter. Infected monolayers were fixed at 12–16 hpi with methanol. MYR2-3xHA was stained with anti-HA antibodies, and GRA7 and MYR1(C) were stained with antibodies to the corresponding recombinant proteins. All else as in (B). **(D)** MYR3 localization. Monolayers were infected with RH tachyzoites stably expressing 3xHA-tagged MYR3, fixed at 12–16 hpi with methanol, and stained as in (B).

*MYR2* is located on chromosome VIII and is predicted by ToxoDB (v32) to have two introns and encode a protein of ~32 kDa, including a predicted N-terminal signal peptide (probability 0.672 by SignalP 2.0 HMM) and, by TMpred, two transmembrane (TM) domains between amino acids 43–60 and 261–279 (Fig 5A). Microarray and RNA-Seq data available in ToxoDB (v32) show that this gene is expressed in tachyzoites and sporozoites (FPKM values of ~50–100) and to a lesser extent in bradyzoites (FPKM and microarray values about 1/10^th^ those seen with tachyzoites) but it is essentially off in the enteroepithelial stages found in felines (FPKM <2). Phosphoproteomics analysis indicates two potential phosphorylation sites on residues 162 and/or 167 [37; ToxoDB v32]. BLAST analysis shows MYR2 has an orthologue in *Neospora caninum* (*NCLIV_036070*) although it shows only 39% amino acid identity to the *Toxoplasma* protein (S1 Fig). Interestingly, the strongest conservation between the two orthologues is in the C-terminal-most 69 amino acids which are 71% identical and this region includes one of the two strongly predicted TM domains. The more N-terminal predicted TM domain (amino acids 43–60) is conserved in overall hydrophobicity and thus is still strongly predicted to be a TM in the *Neospora* sequence, but the exact amino acid sequences in this region are not well conserved. No orthologue of MYR2 is detected in *Sarcocystis* or the more distantly related genus, *Plasmodium*. Unlike MYR1, MYR2 does not appear to have a “RRL” TEXEL motif that would target it for cleavage by ASP5 [18–20].

*MYR3* is located on chromosome X and is annotated by ToxoDB (v32) as having three introns; however, the first and third such predicted introns are spliced out in <1% of the transcripts whereas intron 2 is removed from almost all this gene’s mRNAs. The ORF predicted by removal of just intron 2 encodes a protein of 192 amino acids with no signal peptide but a strongly predicted (by TMpred) TM domain between residues 79–101, which could act as an internal signal sequence to route the protein through the secretory pathway (Figs 5A and S2). RNASeq and microarray data show that *MYR3* is well expressed in tachyzoites and sporozoites (FPKM ~200–800 in both), less so in bradyzoites (FPKM ~60), and essentially off in the enteroepithelial stages (FPKM <1; ToxoDB v32). Interestingly, a few of the non-canonical *Toxoplasma* strains show a deletion of ~250 bp in their 3’-UTR sequence relative to the well-studied RH, ME49 and VEG strains but the impact of this on the gene’s function is not known ([38]; ToxoDB v32). Interestingly, MYR3 shows a high number of spectral counts for phosphorylated serine residues in the context of intracellular infection, but not in the purified parasites, suggesting that the protein is phosphorylated outside of the parasite’s boundaries [37; ToxoDB v32]. BLAST analysis indicated that, like *MYR1* and *MYR2*, *MYR3* has a somewhat conserved orthologue in *Neospora caninum* (*BN1204_051095*) with 35% identity over the N-terminal most 181 amino acids (S2 Fig) and no detectable orthologue in *Plasmodium*. The presence of a predicted TM domain is also conserved in the *Neospora* version of the protein at amino acids 83–99. As with MYR2, MYR3 does not have a recognizable “RRL” TEXEL motif.

### MYR2 and MYR3 co-localize with MYR1 at the PV membrane and space

In a previous study, we showed that MYR1 is essential for effector translocation and is cleaved into two fragments, both of which are found within the PV space and at the PVM [27]. To determine if MYR1 (N)- and (C)-termini precisely co-localize, which was not previously done, we performed confocal immunofluorescence on RH:*MYR1-3xHA* strain, which has a 3xHA tag endogenously fused to the MYR1(C)-terminus, using anti-HA antibodies and antibodies raised to the native MYR1(N) domain to detect the two portions individually. The results show that MYR1 (N) and (C)-termini indeed co-localize at the PV membrane (Fig 5B).

To determine the localization of MYR2, we endogenously modified the gene to express a 3xHA tag either immediately before the stop codon (RH:*MYR2-3xHA*) or after the predicted transmembrane domain, at amino acid residue 62 (RH:*MYR2-3xHA*_*62*_). Although, as discussed further below, both parasite lines yielded a protein product migrating at about 40 kDa by Western blot, neither yielded a visible signal by standard IFA procedures.

The failure to detect the endogenously tagged MYR2 protein by IFA could be due to low protein expression levels. To overcome this, we expressed MYR2-3xHA ectopically off the GRA1 promoter by transient transfection, fixed infected monolayers with methanol, and probed its localization by IFA. The results showed that MYR2 localizes to the PV space and, to some degree, to the PV membrane; it also shows some co-localization with GRA7, a protein known to be localized to the intravacuolar network (IVN) and PVM [10, 39], and with MYR1 in the PV space and membrane (Fig 5C). While these results were obtained with over-expression, they do strongly suggest that MYR2 is IVN- and PVM-associated.

To determine the localization of MYR3, we first generated the RH:*MYR3-3xHA* strain, in which a 3xHA tag is fused endogenously to the 3’-end of the sequence coding for the C-terminus of MYR3, and then used it to infect HFF monolayers. Infected monolayers were fixed with methanol at 12–16 hours, stained with antibodies against HA, MYR1, or GRA7, and then visualized by confocal microscopy. The results indicate that MYR3-3xHA has robust co-localization with GRA7 and MYR1 within the PV and at the PVM (Fig 5D).

Given this localization, we then assessed whether correct localization of either MYR1, MYR2, or MYR3 depends on either of the other MYR proteins. To determine if MYR1 localization depends on MYR2 or MYR3, we infected monolayers with RH, RH*Δmyr2*, or RH*Δmyr3* tachyzoites and stained for MYR1(N) and, as a control, GRA7, using antibodies raised to recombinant versions of these proteins. The results showed that MYR1(N) localizes to the PV and PVM for all three strains with no detectable difference between them (S3 Fig), thereby indicating that MYR1’s localization is not dependent on MYR2 or MYR3.

To determine if MYR2 localization depends on MYR1 or MYR3, we transiently transfected RH*Δmyr1*, RH*Δmyr2*, and RH*Δmyr3* tachyzoites with a construct expressing MYR2-3xHA and assessed MYR2 localization by IFA with HA antibodies. We again found no apparent difference in MYR2-3xHA localization between the three strains (S4 Fig), suggesting that MYR2 localization does not depend on MYR1 or MYR3. To assess MYR3 localization in RH*Δmyr2* parasites, we ectopically integrated *MYR3-3xHA* expressed off its natural promoter in RH and RH*Δmyr2* parasites and stained for MYR3-3xHA with HA antibodies. MYR3-3xHA similarly localized to the PV membrane and space in both strains (S5 Fig), indicating that MYR2 is not necessary for correct localization of MYR3.

### MYR1, MYR2, and MYR3 are membrane-associated outside of the tachyzoite’s boundaries

**Fig 6:**
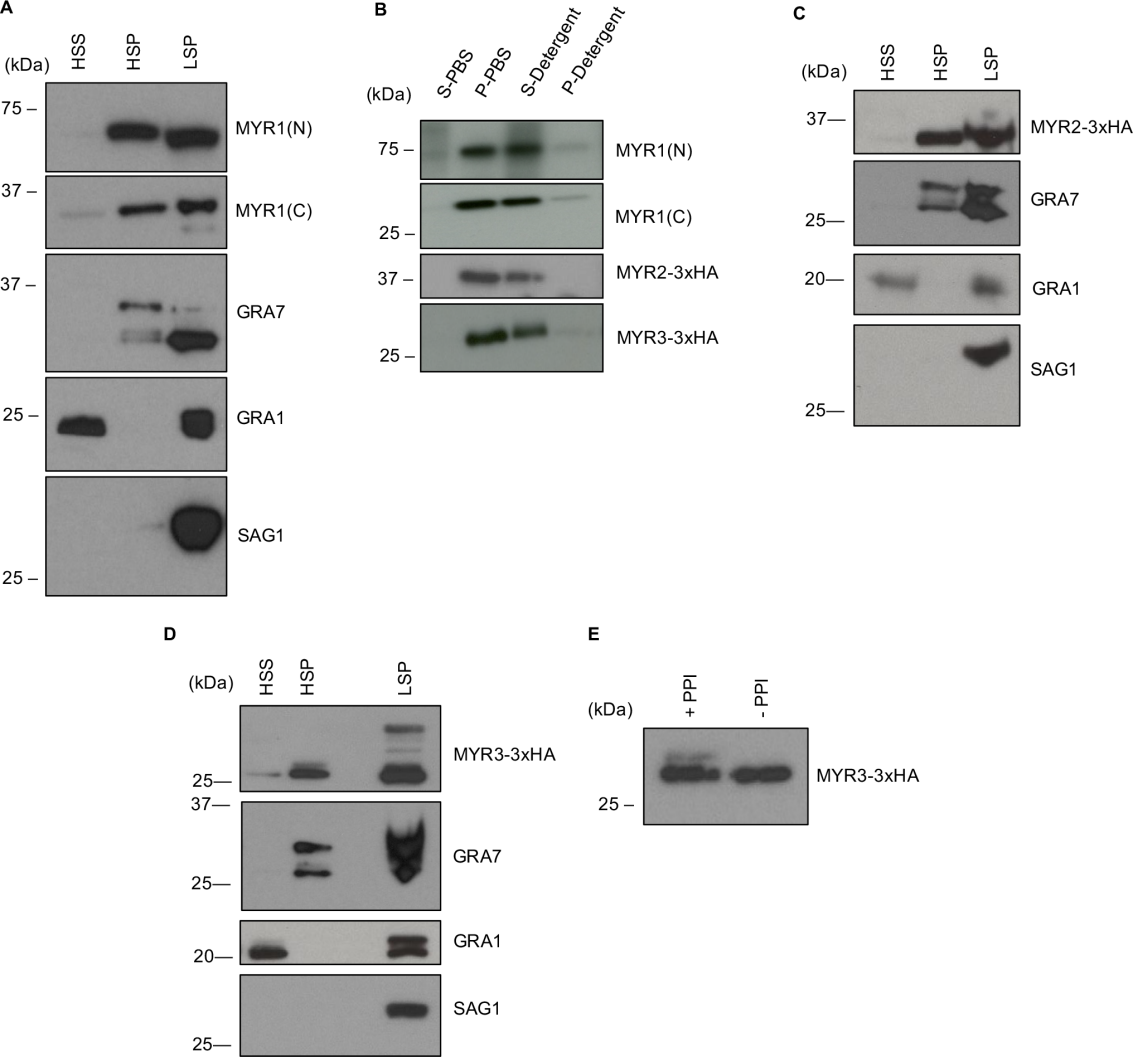
MYR1, MYR2, and MYR3 associate with membranes outside of the parasite. **(A)** Fractionation of MYR1 protein. HFFs were infected with RH *Toxoplasma* tachyzoites for 20–24 hours and then mechanically lysed in PBS with protease and phosphatase inhibitors. Parasites and host cell debris were pelleted at 2500 x g and this resulting low-speed pellet (LSP) was resuspended in lysis buffer, while the supernatant was further fractionated at 100,000 x g. Resulting high-speed supernatant (HSS) and pellet (HSP) as well as the LSP were resolved by SDS-PAGE and blotted with antibodies against MYR1(N) and (C) fragments, GRA7, GRA1 and SAG1. **(B)** The HSP fractions from (A), (D), and (C) were treated with PBS or detergents, fractionated at 100,000 x g, and the resulting supernatant (S-PBS or S-detergent) and pellet (P-PBS or P-detergent) fractions resolved by SDS-PAGE, blotted and probed as in (A). **(C)** As for (A), except using endogenously tagged RH:*MYR2-3xHA*_*62*_ tachyzoites and anti-HA antibody. (**D)** As for (A), except using endogenously tagged RH:*MYR3-3xHA* tachyzoites and anti-HA antibody. **(E)** HFFs were infected with RH:*MYR3-3xHA* tachyzoites and fractionated in PBS supplemented with protease inhibitors plus or minus phosphatase inhibitors (+PPI and -PPI, respectively) according to the protocol described in (A). The resulting HSP fractions were then resolved by SDS-PAGE, blotted and probed with anti-HA antibody.

MYR1, MYR2, and MYR3 are all bioinformatically predicted to have transmembrane domains. Given their localization within the PV and at the PVM (Fig 5), we next asked if they are indeed membrane-associated. To test this, we performed fractionation experiments in which HFFs were infected with the relevant RH tachyzoites for 20–24 hours, mechanically lysed in PBS to release the parasites, and then spun at 2500 x g to pellet intact parasites, host nuclei, and other debris. The resulting pellet, referred to as the low-speed pellet (LSP), was resuspended in lysis buffer, while the supernatant containing PV membranes and host cell material was fractionated at 100,000 x g to isolate the high-speed supernatant (HSS) and high-speed pellet (HSP) fractions. To first determine if MYR1 (C) and (N) are membrane-associated or soluble, we performed SDS-PAGE on the fractions from HFFs infected with RH:*MYR1-3xHA* parasites and blotted for the MYR1 C-terminus with HA antibodies and the MYR1 N-terminus with antibodies raised to the N-terminal domain of MYR1. The fractions were also blotted with antibodies against GRA7, an integral PVM protein [40] and GRA1, a soluble dense granule protein secreted by *Toxoplasma* into the PV space [41], as controls. The results (Fig 6A) showed that, outside of the parasite, both MYR1 (C) and (N) fragments are in the HSP fractions. Importantly, GRA7, shown previously to be within PV membranes [40], is also primarily in the HSP fraction, while GRA1 is in the HSS. To ensure that the pellet fraction consists primarily of PVM membranes and not intact parasites, we blotted with antibodies against SAG1, a GPI-anchored parasite surface antigen. The results showed that, as expected, almost no SAG1 is in the HSP, thereby indicating that MYR1’s presence in this fraction is not a result of contamination with intact parasites.

The presence of protein in the pellet may be the result of membrane association or protein aggregation. To determine whether MYR1 is released by detergents into the supernatant, which is consistent with membrane association, we sonicated the HSP in PBS or detergents, incubated it on ice for 45 minutes, and fractionated again at 100,000 x g. The resulting fractions were then resolved by SDS-PAGE and probed by Western blot. The results showed that both MYR1 fragments are released by detergents into the supernatant, indicating membrane association rather than protein aggregation (Fig 6B).

To determine if MYR2 and MYR3 are also membrane-associated, we performed similar experiments on HFFs infected with RH tachyzoites endogenously HA-tagged at the *MYR2* or *MYR3* locus. The results showed that MYR2 and MYR3 are predominantly in the HSP fraction, not the HSS (Fig 6C and 6D), and the pelleted protein is released by detergents into the supernatant (Fig 6B). The somewhat slower-than-predicted mobility of MYR2 (it migrates as if 40 kDa whereas it is predicted to be about 32 kDa) may be related to its acidic pI (4.7) and/or phosphorylation, both of which can retard a protein’s mobility [42]. Interestingly, MYR3 was detected as a doublet that is most visible in the pellet fraction (Fig 6D). To determine if the doublet is due to phosphorylation, we repeated the fractionation in the presence or absence of phosphatase inhibitors and analyzed the pellet fractions for MYR3-3xHA by Western blot. We found that the slower migrating band was substantially decreased in the absence of phosphatase inhibitors relative to the control (Fig 6E), consistent with this upper band being a phosphorylated form, the existence of which was previously reported in phosphoproteome analyses [37].

### MYR1’s two domains associate in a disulfide-dependent manner

**Fig 7:**
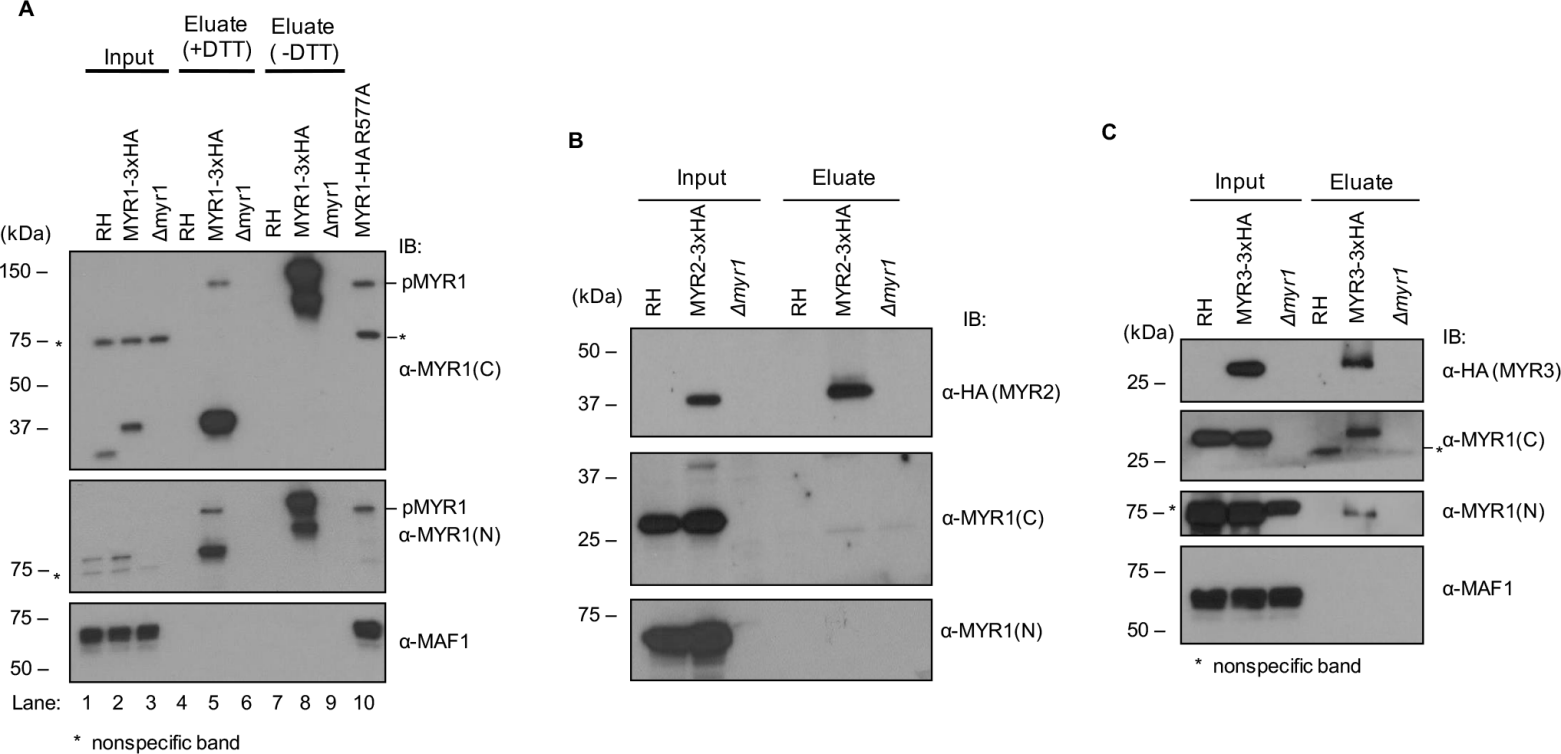
Identification of a MYR1/3 complex. **(A)** Immunoprecipitations were performed using anti-HA magnetic beads incubated with whole lysate from HFFs infected 24 h with the untagged RH strain (lanes 1,4,7), RH:*MYR1-3xHA* strain (lanes 2,5,8), or RH*Δmyr1* strain (lanes 3,6,9). The input contained ~1% of the amount of starting material relative to the eluate lanes. For lanes 7–9, dithiothreitol (DTT) was omitted from the sample buffer to assess disulfide-mediated interactions. Lysate from RH:*MYR1-HA R577A* strain was included as a control (lane 10). Immunoblotting (IB:) was performed with antibodies raised to MYR1(C), MYR1(N) or MAF1 (as a negative control). Size markers in kDa are shown to the left. The asterisk indicates a nonspecific, cross-reacting band that is present even in the *Δmyr1* line, as previously described using these antibodies [27]. pMYR1 indicates the uncleaved precursor MYR1, as previously described [27]. (**B)** Immunoprecipitations were performed as described in (A) except using RH:*MYR2-3xHA*_*62*_ tachyzoites and anti-HA antibodies to detect the HA-tagged MYR2. **(C)** Immunoprecipitations were performed as in (A) except using RH:*MYR3-3xHA* tachyzoites and anti-HA antibodies to detect the HA-tagged MYR3.

As previously shown, MYR1 is cleaved into N- and C-terminal fragments that run at approximately 80 kDa and 32 kDa, respectively [27]. Given that the two fragments co-localize and show similar fractionation patterns, despite MYR1(N) not having a predicted transmembrane domain, we assessed whether the two fragments stably associate. To do this, we performed immunoprecipitations using anti-HA antibodies on HFFs infected 20–24 hpi with RH:*MYR1-3xHA* tachyzoites expressing C-terminally tagged MYR1 or with untagged tachyzoites as a control. The resulting inputs and eluates were boiled in sample buffer containing dithiothreitol (+DTT), resolved by SDS-PAGE, and probed for MYR1(C) and MYR1(N) using antibodies to the corresponding recombinant fragment. The results (Fig 7A) showed that MYR1(N) specifically co-precipitated with MYR1(C), indicating that the two proteins are stably associating (note that the migration of the C-terminal MYR1 domain is somewhat retarded by addition of the triple-HA tag, as expected); no such precipitation was seen using the untagged RH strain as a negative control. To check for specificity, we also blotted for MAF1, an integral membrane protein that localizes to the PV membrane and mediates mitochondrial association [13]. As expected, MAF1 was not co-precipitated (Fig 7A).

To determine whether the interaction between the MYR1(N) and (C) domains is mediated by disulfide bonds (both domains include at least one cysteine residue), we repeated the analysis and excluded DTT from the sample buffer (-DTT). As a positive control for full-length protein, we used a strain with a mutant form of MYR1 (RH:*MYR1-HA R557A*) in which the ASP5-dependent cleavage site is mutated, resulting in unprocessed MYR1 (lane 10). Omitting DTT from the sample buffer resulted in both the N-terminal and C-terminal fragments running at about the size of the uncleaved protein, indicating that N- and C-terminal domains of MYR1 associate in a disulfide-dependent manner (Fig 7A). As previously observed [27], a small amount of uncleaved product was present even in the presence of DTT (lane 5), indicating either incomplete processing by ASP5 or incomplete reduction by DTT.

### MYR3 but not MYR2 appears to associate stably with MYR1

To determine if MYR1 stably interacts with MYR2, we performed immunoprecipitations on HFFs infected with RH:*MYR2-3xHA*_*62*_ and an untagged RH control, as described above. The resulting inputs and eluates were resolved by SDS-PAGE and probed by Western blotting with antibodies to the HA-tag, MYR1(N) or MYR1(C). The results showed no evidence for association of either MYR1 domain with MYR2 (Fig 7B). We cannot rule out that MYR1 and MYR2 interact transiently, or that the apparent lack of an association is an artifact of the conditions used; however, the results suggest that MYR2 does not stably associate with MYR1.

To determine if MYR1 interacts with MYR3, we infected HFFs with RH:*MYR3-3xHA* or untagged RH tachyzoites and repeated the immunoprecipitations and Western blotting described above. Immunoprecipitation from MYR3-3xHA-infected cells is inefficient relative to immunoprecipitations using cells infected with the MYR1-3xHA and MYR2-3xHA-expressing parasites, and does not give a high yield in the eluate. Nevertheless, blotting with HA antibodies showed that some MYR3-3xHA is precipitated in the tagged eluate and that both fragments of MYR1 co-precipitate with MYR3 (Fig 7C). As a control for specificity, we also probed for MAF1 with anti-MAF1 antibodies and saw no detectable MAF1 co-precipitation, confirming that the interaction of MYR3 and MYR1 is specific. Interestingly, the ratio of the precipitated MYR1(C) fragment relative to the input is similar to the ratio for precipitated and input MYR3, suggesting that the interaction could be stoichiometric, although more careful biochemical studies will be needed to conclude this definitively.

### Highly ordered domain blocks effector transport

**Fig 8:**
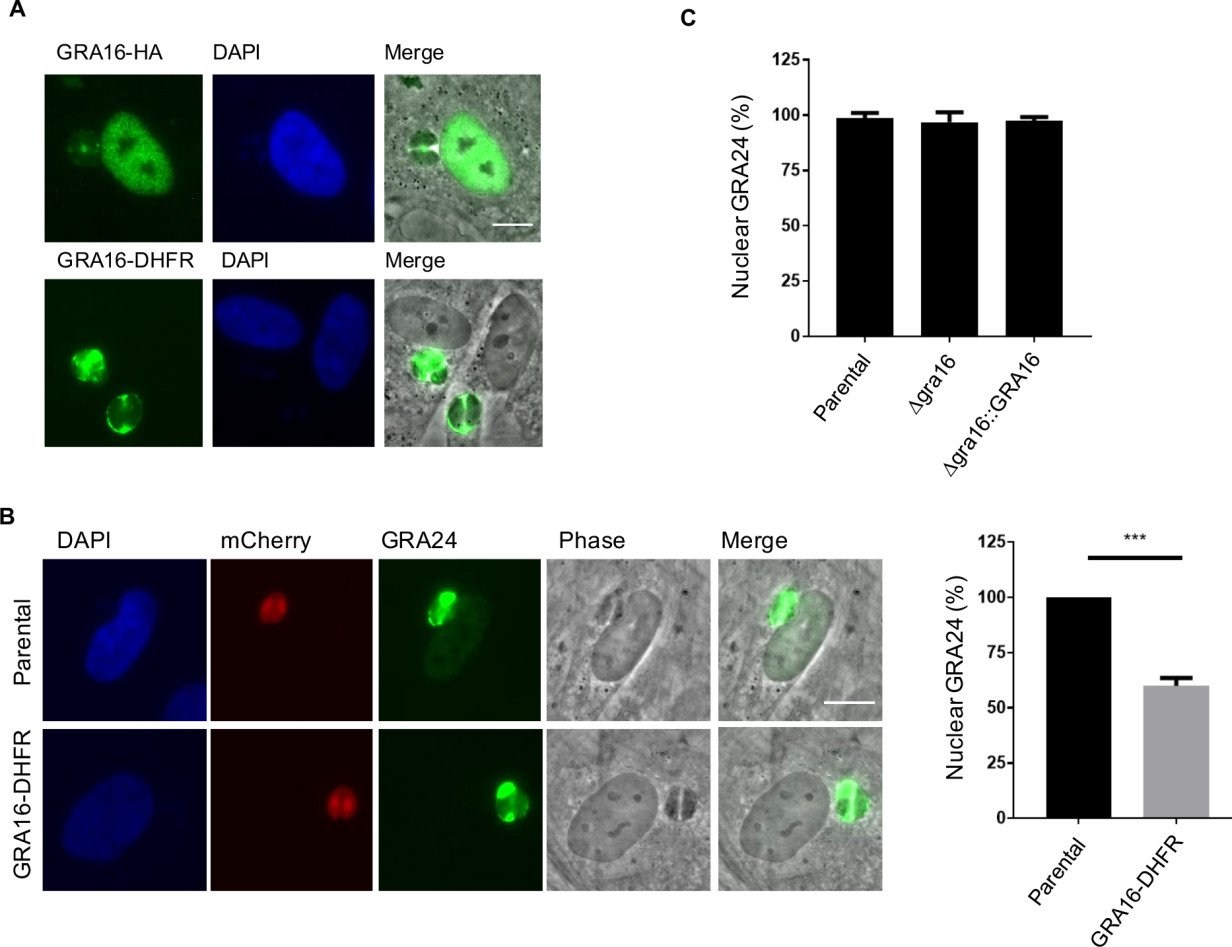
Fusion of GRA16 to DHFR domain blocks effector transport. (A) DHFR domain blocks GRA16 transport to host nucleus. HFFs were infected with RH tachyzoites ectopically expressing GRA16-HA or GRA16-DHFR and fixed at 20 hpi. GRA16-HA was stained with anti-HA antibodies and GRA16-DHFR was stained with anti-DHFR antibodies. (B) GRA16-DHFR expression reduces GRA24 transport to the nucleus. Parental RH and RH::*GRA16-DHFR* tachyzoites were transiently transfected with GRA24-Myc plasmid and fixed at 20–24 hpi. GRA24 was stained with anti-Myc tag antibodies and its localization to the host nucleus was quantified. The averages are based on normalized results from three independent experiments. Error bars indicate standard error of the mean, and statistics were performed with Student’s t-test. *** indicates P < 0.001. (C) GRA24 localization to host nucleus does not depend on GRA16 translocation. HFFs were infected with RH wild type, RH*Δgra16*, and RH*Δgra16*::*GRA16-HA* tachyzoites transiently expressing Myc-tagged GRA24 and fixed at 20–24 hpi. Staining of the host nucleus with antibodies to the Myc-tag is expressed as a percentage of all cells infected with parasites expressing the GRA24 construct. Results are shown for one representative experiment of 3 performed. Scale bar indicates 5 μm.

Three of the *Toxoplasma* effectors that are transported across the PVM, namely GRA16, GRA24, and IST, appear to be highly disordered [11, 15, 16], which may be important for their translocation. Previous work has shown that fusion of GRA16 to DHFR, which rapidly folds into a highly structured domain, blocks GRA16 transport across the PVM [19], even in the absence of domain-stabilizing compounds. To determine if this block on GRA16-DHFR transport affects PVM translocation of other effectors, which would be predicted in a translocon-based model of protein transport, we expressed GRA16-DHFR ectopically and assessed the effect of this fusion on GRA16 and GRA24 transport. In good agreement with previous results, we found that whereas (non-DHFR-fused) GRA16-HA localized to the PV space and trafficked to the host nucleus, GRA16-DHFR accumulated in the vacuole and was rarely visible (< 10%) in the host cell nucleus (Fig 8A). To determine if this block on GRA16-DHFR transport affects translocation of other effectors, such as GRA24, we transiently transfected the parental and GRA16-DHFR-expressing strains with a Myc-tagged GRA24 plasmid and assessed GRA24 localization by IFA. We found that expression of GRA16-DHFR significantly reduced GRA24 localization to the host cell nucleus (Fig 8B), suggesting that either GRA16-DHFR impedes the translocation machinery or that GRA24 directly depends on a fully functional GRA16 for this process, e.g., through some sort of specific association or “piggy-backing”. Notably, unlike GRA16, GRA24 lacks the RRL motif cleaved by ASP5 and does not appear to be cleaved elsewhere, yet still depends on ASP5 for its transport across the PVM [18, 19]. To determine if GRA24 translocation depends on GRA16, we transiently transfected wild type, RH*Δgra16*, and RH*Δgra16*::GRA16-HA tachyzoites with the GRA24-Myc plasmid [18] and assessed GRA24 localization by IFA. The results (Fig 8C) showed no difference in GRA24 translocation between the strains, indicating that GRA24 translocation does not intrinsically depend on GRA16 and that the effect of GRA16-DHFR is through nonspecific blocking of the translocation machinery.

## Discussion

In this study we have identified a novel membrane complex of at least 3 polypeptides—MYR1(N), MYR1(C), and MYR3—that is essential for effector translocation across the vacuolar membrane into the host cell. Specifically, we have identified two novel parasite proteins, MYR2 and MYR3, that are essential for effector translocation and show that they co-localize with GRA7 and MYR1 in the PV space and PVM. Consistent with their predicted transmembrane domains, MYR1, MYR2, and MYR3 all associate with membranes outside of the parasite’s boundaries. Furthermore, co-immunoprecipitation experiments reveal that MYR1 (N)- and (C)-termini stably interact in a disulfide-dependent manner and that MYR3, but not MYR2, stably associates with MYR1, indicating a complex of at least 3 polypeptide chains. It is unclear why MYR1 is cleaved into two fragments that nonetheless associate in a disulfide-dependent manner, but this may play an important regulatory function. Although we did not see any interaction between MYR1 and MYR2, we cannot rule out that these proteins transiently interact and/or that the apparent lack of association is an artifact of the conditions used.

The precise role of MYR1, MYR2 and MYR3 in effector translocation is unclear, but given their localization and membrane association, MYR1 and MYR3 seem likely to form part of a novel complex that directly transports effector proteins across the membrane. The lack of stable association and the low protein abundance of MYR2 relative to MYR1 and MYR3 suggest that MYR2 may function differently, even though all three proteins are clearly essential for GRA16 and GRA24 translocation. Furthermore, MYR1, MYR2, and MYR3 show similar patterns of expression between different developmental stages of *Toxoplasma* based on RNASeq and microarray data, consistent with a coordinated function.

Interestingly, we found that MYR1, MYR2, and MYR3 are not necessary for normal growth within one lytic cycle, but their absence does have a measurable effect on growth over multiple lytic cycles. This suggests that one or more MYR-dependent effectors contributes to the parasite’s ability to grow in HFFs *in vitro*, but the identity of this effector remains to be determined. In our previous work, we showed that mice infected with Type I (RH) *Δmyr1* tachyzoites have a delayed time to death, while mice infected with Type II (ME49) *Δmyr1* tachyzoites show a significant decrease in virulence and survive infection [27]. These results are consistent with the important role that effectors whose translocation is dependent on MYR1, such as GRA16, have been reported to have on virulence [11, 16]. Given that mutants disrupted in *MYR1*, *MYR2* and *MYR3* all have essentially identical phenotypes with regard to *in vitro* growth and defective effector translocation, it is highly likely that they will also show similar virulence phenotypes in mice.

Like MYR1, clear orthologues of MYR2 and MYR3 are present in *Neospora caninum*, consistent with their function as a translocon. Surprisingly, however, the level of conservation between the two genera for all three proteins is modest: 45%, 39% and 35% for MYR1, MYR2 and MYR3, respectively. This suggests that the translocation machinery has been subject to selection for optimal efficiency with their respective cargoes. Interestingly, none of these MYR proteins have homologues in *Plasmodium* and, similarly, the PTEX machinery that translocates secreted *Plasmodium* proteins across the PVM in an infected red blood cell is not functionally conserved in *Toxoplasma;* while there are two homologs of PfEXP2 in *Toxoplasma*, GRA17 and GRA23, these have recently been shown to function in the translocation of small molecules (<1800 daltons) across the PVM, not proteins [26].

The requirements for translocation across *Toxoplasma*’s PVM are not well understood, but they appear to differ from those for *Plasmodium*. Fusion of PTEX substrates in *Plasmodium* to highly ordered domains such as DHFR and GFP does not significantly affect their transport unless a stabilizing molecule that prevents their unfolding is added; however, for *Toxoplasma*, addition of a highly structured domain, such as DHFR, to the GRA16 effector is sufficient to block export even in the absence of additional stabilizing compounds, as reported here and elsewhere [19, 43]. Importantly, the fusion of GRA16 to DHFR also impedes the translocation of GRA24; this is similar to the “blockage” effects that have been reported for protein translocons in other systems [44] and is consistent with the existence of a translocon at the PVM rather than a less constrained mechanism for protein translocation across this membrane. Overall, our results show that a novel membrane protein complex is essential for translocation and is at the right time and place to be the PVM translocon; however, further work will be needed to definitively determine if the complex operates in this role or as part of an accessory complex that functions upstream or downstream of the actual translocon.

## Supporting information

S1 File Primers, plasmids, and strains generated in this study. (XLSX)

S1 Fig Sequence alignment of MYR2 protein. The amino acid sequences of MYR2 in *Toxoplasma gondii* strains ME49, MAS, RH, VEG and RUB were aligned to each other and to the MYR2 orthologs in *Neospora caninum* and *Hammondia hammon*di by Clustal Omega. The residue color indicates the following: red, hydrophobic; blue, acidic; magenta, basic; green, hydroxyl or sulfhydryl or amine group. Predicted signal peptide (SP) and transmembrane domains (TM) are shown. Asterisk (*) indicates fully conserved residue. Colon (:) indicates conservation of strongly similar properties (> 0.5 in the Gonnet PAM 250 matrix). Period (.) indicates conservation of weakly similar properties (< 0.5 and > 0 in Gonnet PAM 250 matrix).
(TIF)

S2 Fig Sequence alignment of MYR3 protein. The amino acid sequences of MYR3 in *Toxoplasma gondii* strains ME49, MAS, RH, VEG and RUB were aligned to each other and to the MYR3 orthologs in *Neospora caninum* and *Hammondia hammondi* by Clustal Omega. All other details as in S1 Fig.
(TIF)

S3 Fig MYR1(N) localizes to the PV membrane and space in the absence of MYR2 or MYR3. HFFs were infected with RH, RH*Δmyr1*, RH*Δmyr2*, or RH*Δmyr3* tachyzoites for 18 h and fixed with methanol. MYR1(N) and GRA7 were stained with antibodies to the respective recombinant protein and their localization assessed by confocal microscopy as described in (5B). Note that there is a low level of staining with the anti-MYR1 antibody within the parasite cytoplasm in the RH*Δmyr1* parasites but this does not interfere with the MYR1-specific signal at the PVM seen in the other strains. Scale bar indicates 5 μm.
(TIF)

S4 Fig MYR2 localizes to the PV membrane and space in the absence of MYR1 or MYR3. RH*Δmyr1*, RH*Δmyr2*, and RH*Δmyr3* tachyzoites were transiently transfected with a construct that expresses 3xHA-tagged MYR2 off the *GRA1* promoter. Infected monolayers were fixed at 12–16 hpi with methanol and stained and visualized as described in (5C). Scale bar indicates 5 μm.
(TIF)

S5 Fig MYR3 localizes to the PV membrane and space in the absence of MYR2. RH::*MYR3-3xHA* and RH*Δmyr2*::*MYR3-3xHA* parasites were allowed to infect HFFs for 16 before fixation with methanol. MYR3-3xHA was stained with anti-HA antibodies. All else was performed as described in (5B).
(TIF)

## References

[1] Dubey J.P., Advances in the life cycle of Toxoplasma gondii. Int J Parasitol, 1998 28(7): p. 1019–24. 9724872 10.1016/s0020-7519(98)00023-x

[2] Morisaki J.H., Heuser J.E., and Sibley L.D., Invasion of Toxoplasma gondii occurs by active penetration of the host cell. J Cell Sci, 1995 108 (Pt 6): p. 2457–64. 7673360 10.1242/jcs.108.6.2457

[3] Martens S., et al, Disruption of Toxoplasma gondii parasitophorous vacuoles by the mouse p47-resistance GTPases. PLoS Pathog, 2005 1(3): p. e24 doi: 10.1371/journal.ppat.0010024 16304607 10.1371/journal.ppat.0010024 PMC1287907

[4] Melzer T., et al, The gamma interferon (IFN-gamma)-inducible GTP-binding protein IGTP is necessary for toxoplasma vacuolar disruption and induces parasite egression in IFN-gamma-stimulated astrocytes. Infect Immun, 2008 76(11): p. 4883–94. doi: 10.1128/IAI.01288-07 18765738 10.1128/IAI.01288-07 PMC2573374

[5] Rosowski E.E., et al, Strain-specific activation of the NF-kappaB pathway by GRA15, a novel Toxoplasma gondii dense granule protein. J Exp Med, 2011 208(1): p. 195–212. doi: 10.1084/jem.20100717 21199955 10.1084/jem.20100717 PMC3023140

[6] Saeij J.P., et al, Polymorphic secreted kinases are key virulence factors in toxoplasmosis. Science, 2006 314(5806): p. 1780–3. doi: 10.1126/science.1133690 17170306 10.1126/science.1133690 PMC2646183

[7] Saeij J.P., et al, Toxoplasma co-opts host gene expression by injection of a polymorphic kinase homologue. Nature, 2007 445(7125): p. 324–7. doi: 10.1038/nature05395 17183270 10.1038/nature05395 PMC2637441

[8] Behnke M.S., et al, The polymorphic pseudokinase ROP5 controls virulence in Toxoplasma gondii by regulating the active kinase ROP18. PLoS Pathog, 2012 8(11): p. e1002992 doi: 10.1371/journal.ppat.1002992 23144612 10.1371/journal.ppat.1002992 PMC3493473

[9] El Hajj H., et al, ROP18 is a rhoptry kinase controlling the intracellular proliferation of Toxoplasma gondii. PLoS Pathog, 2007 3(2): p. e14 doi: 10.1371/journal.ppat.0030014 17305424 10.1371/journal.ppat.0030014 PMC1797617

[10] Etheridge R.D., et al, The Toxoplasma pseudokinase ROP5 forms complexes with ROP18 and ROP17 kinases that synergize to control acute virulence in mice. Cell Host Microbe, 2014 15(5): p. 537–50. doi: 10.1016/j.chom.2014.04.002 24832449 10.1016/j.chom.2014.04.002 PMC4086214

[11] Bougdour A., et al, Host cell subversion by Toxoplasma GRA16, an exported dense granule protein that targets the host cell nucleus and alters gene expression. Cell Host Microbe, 2013 13(4): p. 489–500. doi: 10.1016/j.chom.2013.03.002 23601110 10.1016/j.chom.2013.03.002

[12] Franco M., Shastri A.J., and Boothroyd J.C., Infection by Toxoplasma gondii specifically induces host c-Myc and the genes this pivotal transcription factor regulates. Eukaryot Cell, 2014 13(4): p. 483–93. doi: 10.1128/EC.00316-13 24532536 10.1128/EC.00316-13 PMC4000098

[13] Pernas L., et al, Toxoplasma effector MAF1 mediates recruitment of host mitochondria and impacts the host response. PLoS Biol, 2014 12(4): p. e1001845 doi: 10.1371/journal.pbio.1001845 24781109 10.1371/journal.pbio.1001845 PMC4004538

[14] Ma J.S., et al, Selective and strain-specific NFAT4 activation by the Toxoplasma gondii polymorphic dense granule protein GRA6. J Exp Med, 2014 211(10): p. 2013–32. doi: 10.1084/jem.20131272 25225460 10.1084/jem.20131272 PMC4172224

[15] Braun L., et al, A Toxoplasma dense granule protein, GRA24, modulates the early immune response to infection by promoting a direct and sustained host p38 MAPK activation. J Exp Med, 2013 210(10): p. 2071–86. doi: 10.1084/jem.20130103 24043761 10.1084/jem.20130103 PMC3782045

[16] Olias P., et al, Toxoplasma Effector Recruits the Mi-2/NuRD Complex to Repress STAT1 Transcription and Block IFN-gamma-Dependent Gene Expression. Cell Host Microbe, 2016 20(1): p. 72–82. doi: 10.1016/j.chom.2016.06.006 27414498 10.1016/j.chom.2016.06.006 PMC4947229

[17] Gay G., et al, Toxoplasma gondii TgIST co-opts host chromatin repressors dampening STAT1-dependent gene regulation and IFN-gamma-mediated host defenses. J Exp Med, 2016 213(9): p. 1779–98. doi: 10.1084/jem.20160340 27503074 10.1084/jem.20160340 PMC4995087

[18] Coffey M.J., et al, An aspartyl protease defines a novel pathway for export of Toxoplasma proteins into the host cell. Elife, 2015 4. 10.7554/eLife.10809 PMC4764566 26576949

[19] Curt-Varesano A., et al, The aspartyl protease TgASP5 mediates the export of the Toxoplasma GRA16 and GRA24 effectors into host cells. Cell Microbiol, 2016 18(2): p. 151–67. doi: 10.1111/cmi.12498 26270241 10.1111/cmi.12498

[20] Hammoudi P.M., et al, Fundamental Roles of the Golgi-Associated Toxoplasma Aspartyl Protease, ASP5, at the Host-Parasite Interface. PLoS Pathog, 2015 11(10): p. e1005211 doi: 10.1371/journal.ppat.1005211 26473595 10.1371/journal.ppat.1005211 PMC4608785

[21] Pellegrini E., et al, Structural Basis for the Subversion of MAP Kinase Signaling by an Intrinsically Disordered Parasite Secreted Agonist. Structure, 2017 25(1): p. 16–26. doi: 10.1016/j.str.2016.10.011 27889209 10.1016/j.str.2016.10.011 PMC5222587

[22] Beck J.R., et al, PTEX component HSP101 mediates export of diverse malaria effectors into host erythrocytes. Nature, 2014 511(7511): p. 592–5. doi: 10.1038/nature13574 25043010 10.1038/nature13574 PMC4130291

[23] de Koning-Ward T.F., et al, A newly discovered protein export machine in malaria parasites. Nature, 2009 459(7249): p. 945–9. doi: 10.1038/nature08104 19536257 10.1038/nature08104 PMC2725363

[24] Elsworth B., et al, PTEX is an essential nexus for protein export in malaria parasites. Nature, 2014 511(7511): p. 587–91. doi: 10.1038/nature13555 25043043 10.1038/nature13555

[25] Gehde N., et al, Protein unfolding is an essential requirement for transport across the parasitophorous vacuolar membrane of Plasmodium falciparum. Mol Microbiol, 2009 71(3): p. 613–28. doi: 10.1111/j.1365-2958.2008.06552.x 19040635 10.1111/j.1365-2958.2008.06552.x

[26] Gold D.A., et al, The Toxoplasma Dense Granule Proteins GRA17 and GRA23 Mediate the Movement of Small Molecules between the Host and the Parasitophorous Vacuole. Cell Host Microbe, 2015 17(5): p. 642–52. doi: 10.1016/j.chom.2015.04.003 25974303 10.1016/j.chom.2015.04.003 PMC4435723

[27] Franco M., et al, A Novel Secreted Protein, MYR1, Is Central to Toxoplasma's Manipulation of Host Cells. MBio, 2016 7(1): p. e02231–15. doi: 10.1128/mBio.02231-15 26838724 10.1128/mBio.02231-15 PMC4742717

[28] Fox B.A., et al, Efficient gene replacements in Toxoplasma gondii strains deficient for nonhomologous end joining. Eukaryot Cell, 2009 8(4): p. 520–9. doi: 10.1128/EC.00357-08 19218423 10.1128/EC.00357-08 PMC2669201

[29] Langmead B. and Salzberg S.L., Fast gapped-read alignment with Bowtie 2. Nat Methods, 2012 9(4): p. 357–9. doi: 10.1038/nmeth.1923 22388286 10.1038/nmeth.1923 PMC3322381

[30] McKenna A., et al, The Genome Analysis Toolkit: a MapReduce framework for analyzing next-generation DNA sequencing data. Genome Res, 2010 20(9): p. 1297–303. doi: 10.1101/gr.107524.110 20644199 10.1101/gr.107524.110 PMC2928508

[31] Li H., et al, The Sequence Alignment/Map format and SAMtools. Bioinformatics, 2009 25(16): p. 2078–9. doi: 10.1093/bioinformatics/btp352 19505943 10.1093/bioinformatics/btp352 PMC2723002

[32] Cingolani P., et al, A program for annotating and predicting the effects of single nucleotide polymorphisms, SnpEff: SNPs in the genome of Drosophila melanogaster strain w1118; iso-2; iso-3. Fly (Austin), 2012 6(2): p. 80–92. 10.4161/fly.19695 PMC3679285 22728672

[33] Brymora A., Valova V.A., and Robinson P.J., Protein-protein interactions identified by pull-down experiments and mass spectrometry. Curr Protoc Cell Biol, 2004. Chapter 17: p. Unit 17 5. 10.1002/0471143030.cb1705s22 18228443

[34] Dunn J.D., et al, The Toxoplasma gondii dense granule protein GRA7 is phosphorylated upon invasion and forms an unexpected association with the rhoptry proteins ROP2 and ROP4. Infect Immun, 2008 76(12): p. 5853–61. doi: 10.1128/IAI.01667-07 18809661 10.1128/IAI.01667-07 PMC2583583

[35] Shen B., et al, Efficient gene disruption in diverse strains of Toxoplasma gondii using CRISPR/CAS9. MBio, 2014 5(3): p. e01114–14. doi: 10.1128/mBio.01114-14 24825012 10.1128/mBio.01114-14 PMC4030483

[36] Caffaro C.E., et al, A nucleotide sugar transporter involved in glycosylation of the Toxoplasma tissue cyst wall is required for efficient persistence of bradyzoites. PLoS Pathog, 2013 9(5): p. e1003331 doi: 10.1371/journal.ppat.1003331 23658519 10.1371/journal.ppat.1003331 PMC3642066

[37] Treeck M., et al, The phosphoproteomes of Plasmodium falciparum and Toxoplasma gondii reveal unusual adaptations within and beyond the parasites' boundaries. Cell Host Microbe, 2011 10(4): p. 410–9. doi: 10.1016/j.chom.2011.09.004 22018241 10.1016/j.chom.2011.09.004 PMC3254672

[38] Minot S., et al, Admixture and recombination among Toxoplasma gondii lineages explain global genome diversity. Proc Natl Acad Sci U S A, 2012 109(33): p. 13458–63. doi: 10.1073/pnas.1117047109 22847430 10.1073/pnas.1117047109 PMC3421188

[39] Coppens I., et al, Toxoplasma gondii sequesters lysosomes from mammalian hosts in the vacuolar space. Cell, 2006 125(2): p. 261–74. doi: 10.1016/j.cell.2006.01.056 16630815 10.1016/j.cell.2006.01.056

[40] Neudeck A., et al, Expression variance, biochemical and immunological properties of Toxoplasma gondii dense granule protein GRA7. Microbes Infect, 2002 4(6): p. 581–90. 12048027 10.1016/s1286-4579(02)01576-9

[41] Cesbron-Delauw M.F., Dense-granule organelles of Toxoplasma gondii: their role in the host-parasite relationship. Parasitol Today, 1994 10(8): p. 293–6. 15275422 10.1016/0169-4758(94)90078-7

[42] Shirai A., et al, Global analysis of gel mobility of proteins and its use in target identification. J Biol Chem, 2008 283(16): p. 10745–52. doi: 10.1074/jbc.M709211200 18292091 10.1074/jbc.M709211200

[43] Hakimi M.A., Olias P., and Sibley L.D., Toxoplasma Effectors Targeting Host Signaling and Transcription. Clin Microbiol Rev, 2017 30(3): p. 615–645. doi: 10.1128/CMR.00005-17 28404792 10.1128/CMR.00005-17 PMC5475222

[44] Amyot W.M., deJesus D., and Isberg R.R., Poison domains block transit of translocated substrates via the Legionella pneumophila Icm/Dot system. Infect Immun, 2013 81(9): p. 3239–52. doi: 10.1128/IAI.00552-13 23798536 10.1128/IAI.00552-13 PMC3754216

