# Supplementary figures and images for: Identification of a novel protein complex essential for effector translocation across the parasitophorous vacuole membrane of Toxoplasma gondii
Source: PLoS Pathog. 2018 Jan 22;14(1):e1006828. doi: 10.1371/journal.ppat.1006828 (PMC5794187; doi:10.1371/journal.ppat.1006828)

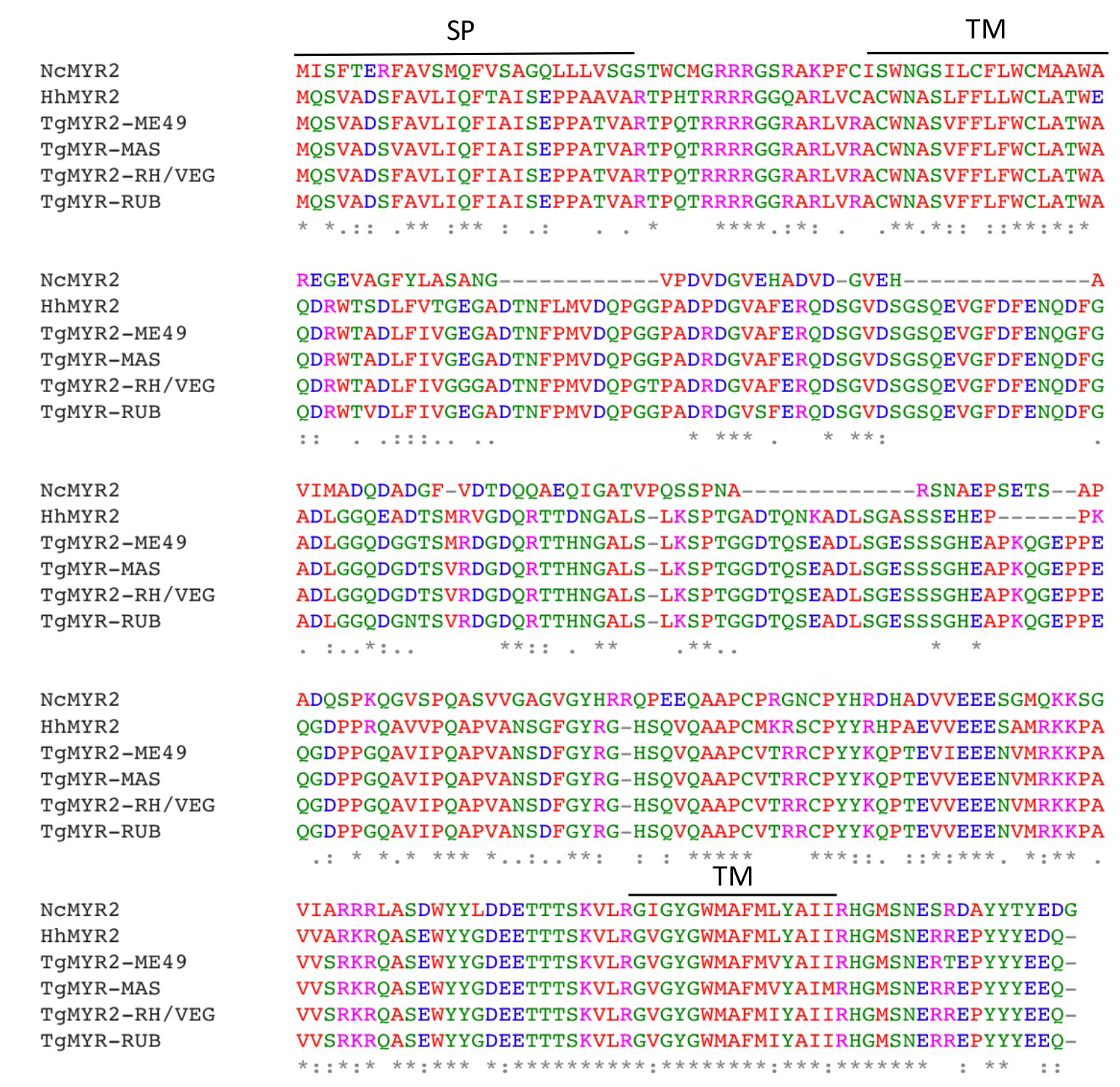

Supplement: S1 Fig — The amino acid sequences of MYR2 in Toxoplasma gondii strains ME49, MAS, RH, VEG and RUB were aligned to each other and to the MYR2 orthologs in Neospora caninum and Hammondia hammondi by Clustal Omega. The residue color indicates the following: red, hydrophobic; blue, acidic; magenta, basic; green, hydroxyl or sulfhydryl or amine group. Predicted signal peptide (SP) and transmembrane domains (TM) are shown. Asterisk (*) indicates fully conserved residue. Colon (:) indicates conservation of strongly similar properties (> 0.5 in the Gonnet PAM 250 matrix). Period (.) indicates conservation of weakly similar properties (< 0.5 and > 0 in Gonnet PAM 250 matrix). (TIF) [file ppat.1006828.s002.tif]

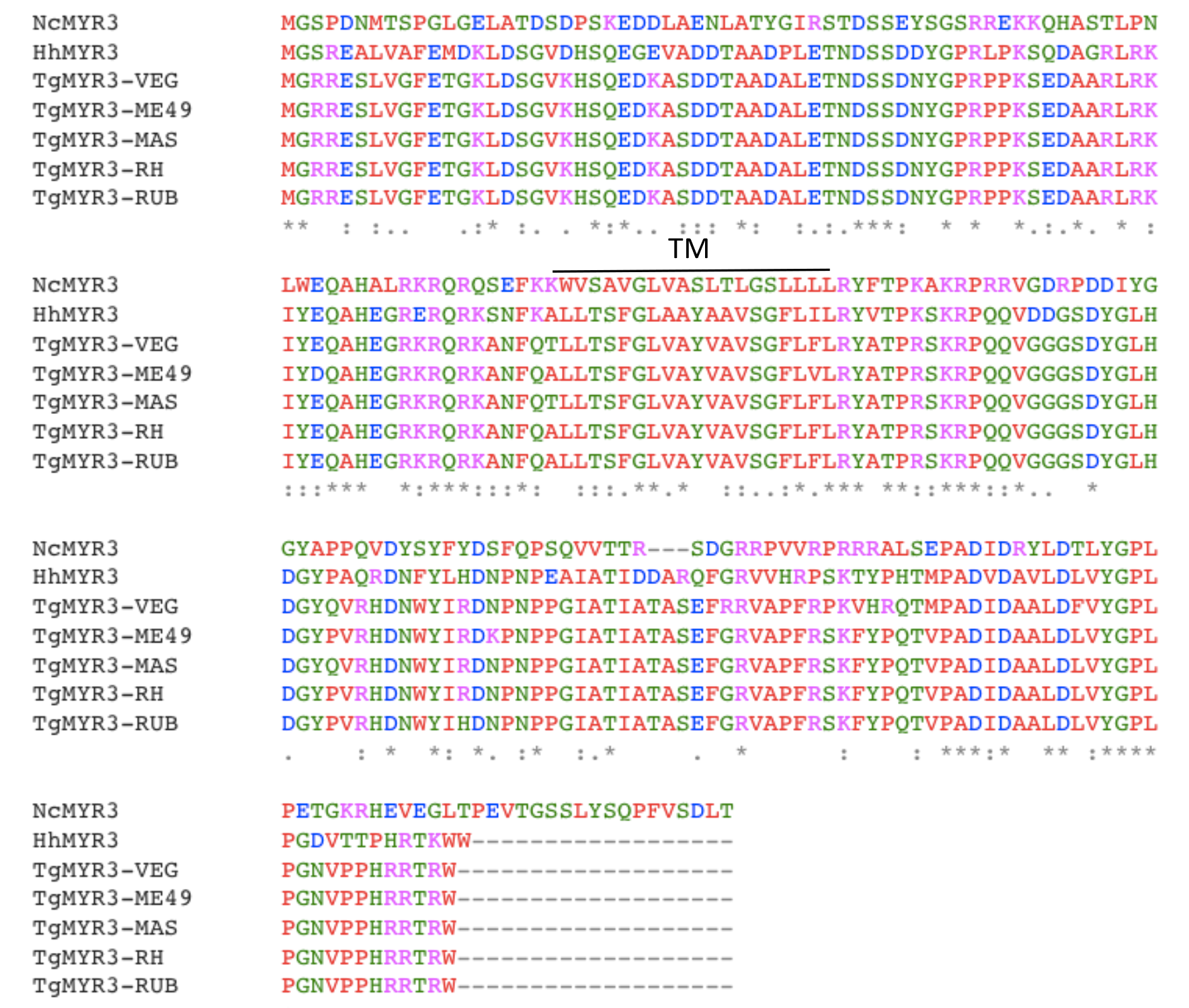

Supplement: S2 Fig — The amino acid sequences of MYR3 in Toxoplasma gondii strains ME49, MAS, RH, VEG and RUB were aligned to each other and to the MYR3 orthologs in Neospora caninum and Hammondia hammondi by Clustal Omega. All other details as in S1 Fig. (TIF) [file ppat.1006828.s003.tif]

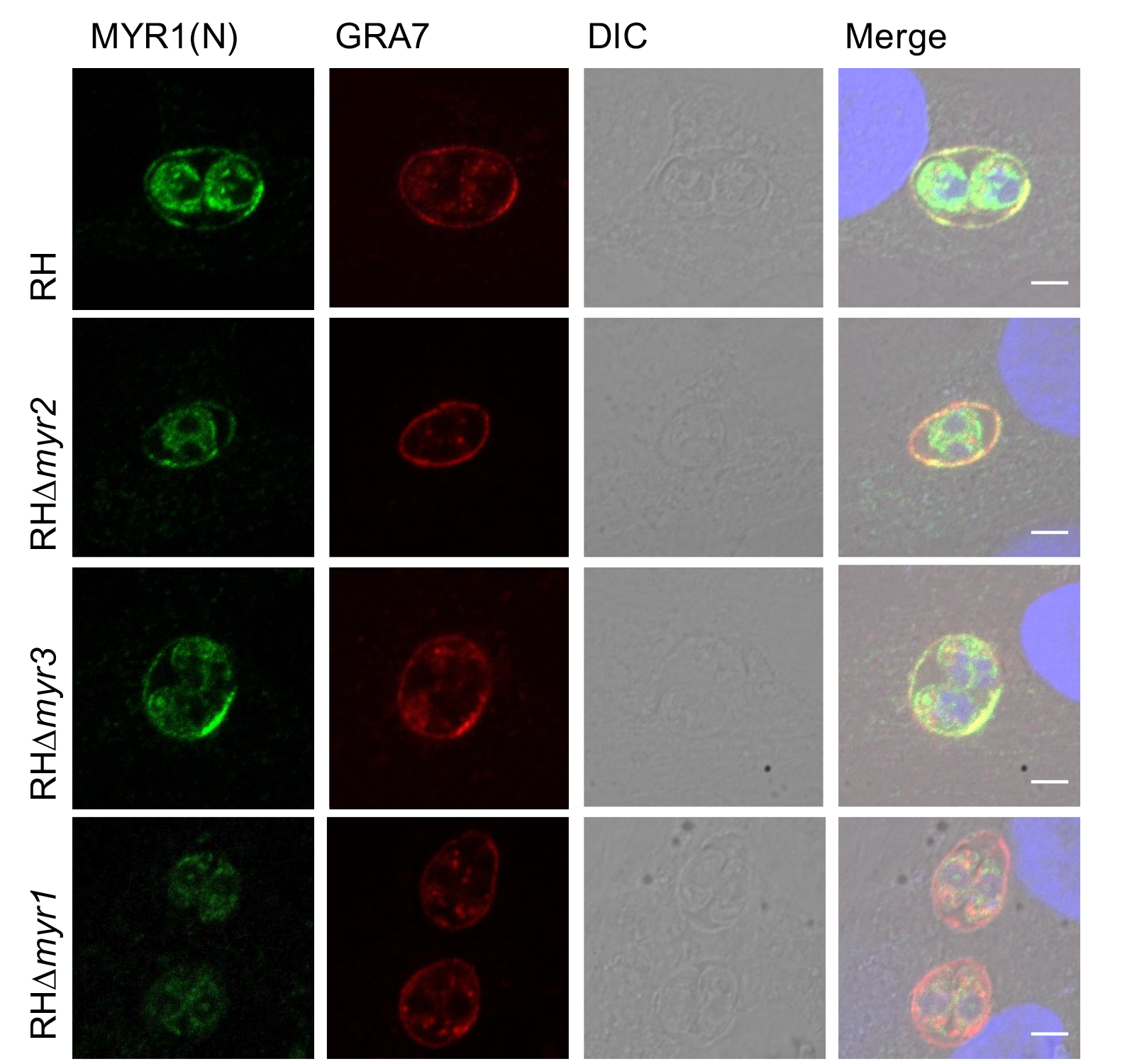

Supplement: S3 Fig — HFFs were infected with RH, RHΔmyr1, RHΔmyr2, or RHΔmyr3 tachyzoites for 18 h and fixed with methanol. MYR1(N) and GRA7 were stained with antibodies to the respective recombinant protein and their localization assessed by confocal microscopy as described in (5B). Note that there is a low level of staining with the anti-MYR1 antibody within the parasite cytoplasm in the RHΔmyr1 parasites but this does not interfere with the MYR1-specific signal at the PVM seen in the other strains. Scale bar indicates 5 μm. (TIF) [file ppat.1006828.s004.tif]

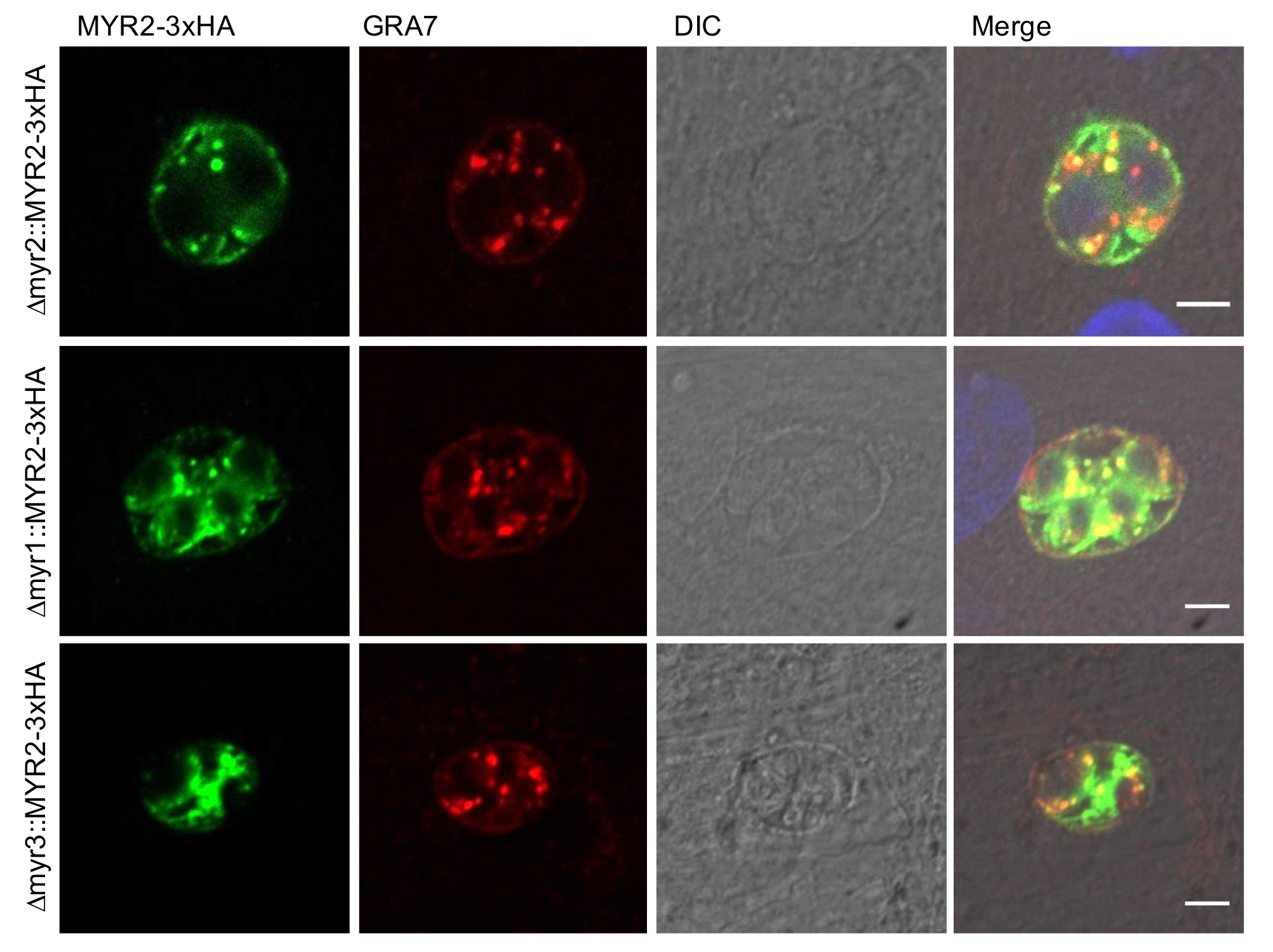

Supplement: S4 Fig — RHΔmyr1, RHΔmyr2, and RHΔmyr3 tachyzoites were transiently transfected with a construct that expresses 3xHA-tagged MYR2 off the GRA1 promoter. Infected monolayers were fixed at 12–16 hpi with methanol and stained and visualized as described in (5C). Scale bar indicates 5 μm. (TIF) [file ppat.1006828.s005.tif]

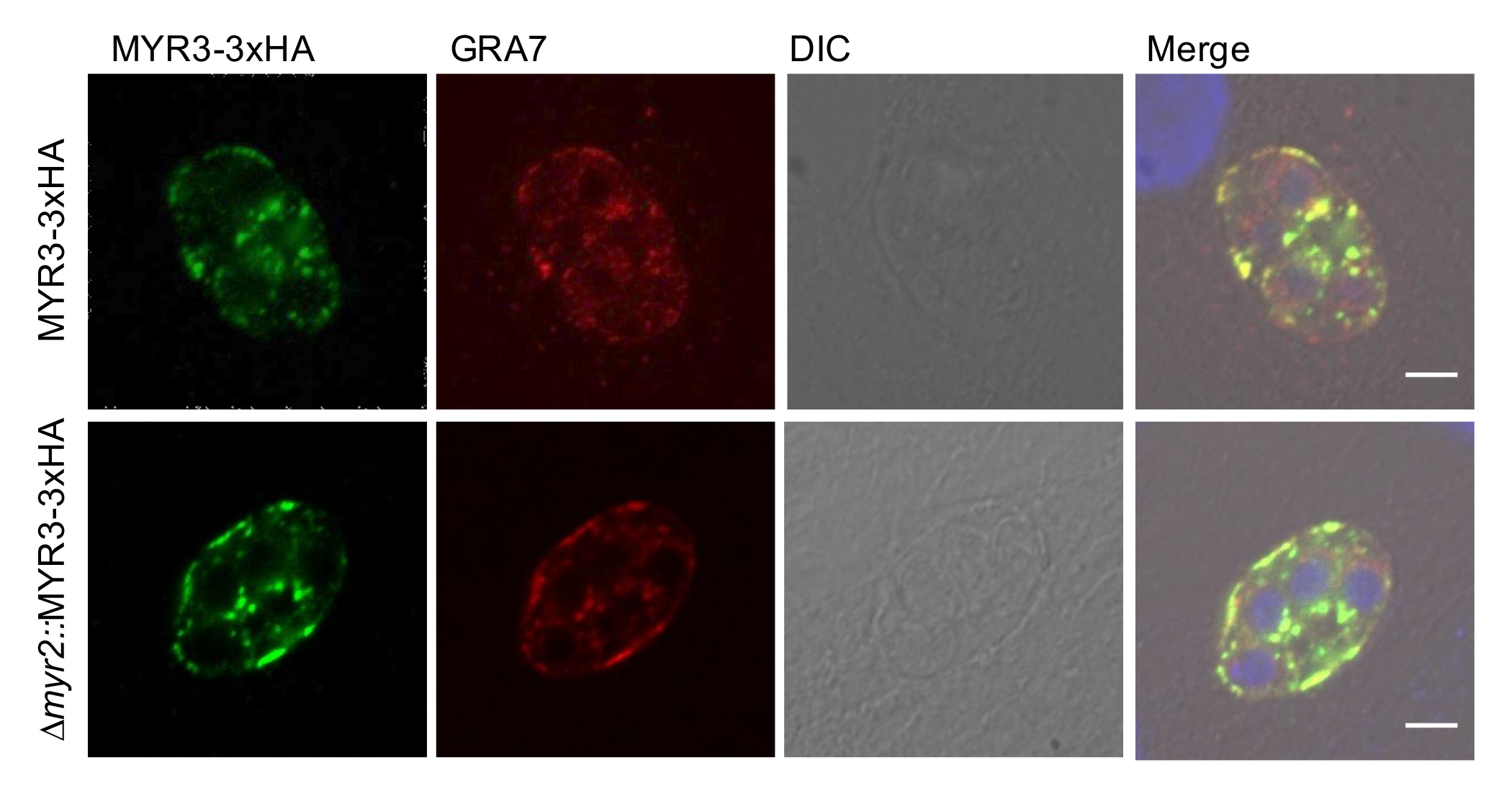

Supplement: S5 Fig — RH::MYR3-3xHA and RHΔmyr2::MYR3-3xHA parasites were allowed to infect HFFs for 16 before fixation with methanol. MYR3-3xHA was stained with anti-HA antibodies. All else was performed as described in (5B). (TIF) [file ppat.1006828.s006.tif]
